# m^6^A‐modified DRAM1 recognized by YTHDF1 regulates autophagy during dexamethasone‐induced osteogenic inhibition

**DOI:** 10.1002/ctm2.70655

**Published:** 2026-07-22

**Authors:** Ze‐Yu Lu, Peng‐Bo Chen, Qing‐Yin Xu, Muradil Mardan, Yue‐Hua Yang, Tao Liu, Wen‐Ning Xu, Huo‐Liang Zheng, Hao Cai, Hui Deng, Xing‐Xu Huang, Bo Li, Sheng‐Dan Jiang, Lei‐Sheng Jiang, Xin‐Feng Zheng

**Affiliations:** ^1^ Spine Center Xinhua Hospital, Shanghai Jiao Tong University School of Medicine Shanghai China; ^2^ Department of Orthopedic Surgery Xinhua Hospital, Shanghai Jiao Tong University School of Medicine Shanghai China; ^3^ Department of Orthopedic Surgery The Affiliated People's Hospital of Jiangsu University Zhenjiang China; ^4^ Department of Spinal Surgery Orthopedic Medical Center Zhujiang Hospital, Southern Medical University Guangzhou China; ^5^ School of Life Science and Technology ShanghaiTech University Shanghai China

**Keywords:** autophagy, dexamethasone, N^6^‐methyladenosine, osteogenic differentiation, Wnt/β‐catenin signalling

## Abstract

Bone health is critically dependent on the precise regulation of bone‐forming cells, which are significantly impacted by glucocorticoids. Although DNA damage‐regulated autophagy modulator 1 (DRAM1) plays a key role in autophagy, its involvement in glucocorticoid‐induced osteoporosis has not been fully explored. Here, we showed that alterations in N^6^‐methyladenosine (m^6^A) levels contributed to the regulation of DRAM1 under dexamethasone treatment. In vitro, our research revealed that high doses of dexamethasone impaired the m^6^A‐dependent regulation of DRAM1 by YTH m^6^A RNA binding protein F1 (YTHDF1), leading to decreased DRAM1 levels and subsequent disruption of autophagy‐associated osteogenic differentiation in human bone marrow mesenchymal stem cells (hBMSCs) and MC3T3‐E1 cells. Additionally, the classic Wnt/β‐catenin pathway, which plays a critical role in bone formation, was shown to be modulated by DRAM1 during osteogenic differentiation. In vivo experiments showed that DRAM1 protein expression in the femurs of Ythdf1 knockout (KO) mice was significantly lower than that in the wild‐type (WT) group, and that resveratrol not only mitigated dexamethasone‐induced bone damage but was also associated with increased DRAM1 expression. These findings advance our understanding of how glucocorticoids hinder bone formation and suggest that targeting the m^6^A‐dependent YTHDF1/DRAM1 regulatory axis may offer novel strategies for osteoporosis treatment.

## Highlights

- High‐dose dexamethasone suppresses YTHDF1 expression and inhibits YTHDF1‐mediated DRAM1 translation.
- YTHDF1 promotes DRAM1 translation through an m^6^A‐dependent mechanism.
- DRAM1 regulates autophagic activity via AKT/rpS6 signaling pathway.
- DRAM1 modulates osteogenesis through Wnt/β‐catenin signaling.
- Targeting the YTHDF1/DRAM1 axis represents a potential therapeutic strategy for glucocorticoid‐induced osteoporosis.

## Graphical abstract

- DRAM1 plays a critical role in the modulation of autophagy.
- The m^6^A methylation level of DRAM1 mRNA is suppressed by dexamethasone.
- Dexamethasone impairs autophagy‐associated osteogenic differentiation by inhibiting YTHDF1‐mediated translation of m^6^A‐modified DRAM1.

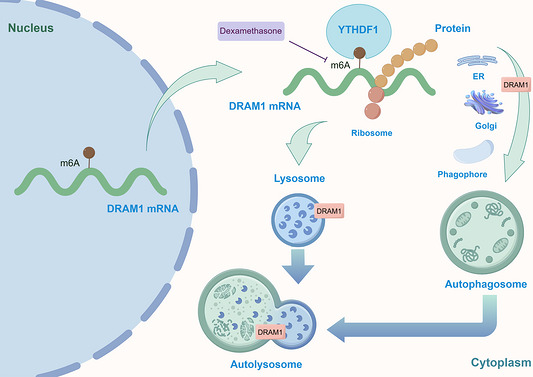

## 1 INTRODUCTION

Glucocorticoids (GCs) are widely used anti‐inflammatory and immunosuppressive drugs for various clinical conditions. However, long‐term or high‐dose GC use can negatively affect osteoblasts and bone marrow mesenchymal stem cells (BMSCs) by reducing their viability, proliferation and differentiation, ultimately leading to osteoporosis.^1^, ^2^ Glucocorticoid‐induced osteoporosis (GIOP) is one of the most common forms of secondary osteoporosis, characterized by decreased bone mass, impaired bone quality and increased fracture risk.^3^ These pathological changes are closely associated with reduced autophagy.^4^ Excessive GCs inhibit autophagy in osteoblasts, as evidenced by decreased levels of markers such as LC3, Beclin1 and ATGs.^5^, ^6^ Conversely, in vivo studies have shown that compounds such as vitamin K2, geniposide and resveratrol can alleviate GIOP by activating autophagy.^7^, ^8^, ^9^ These findings suggest that enhancing autophagy may represent a potential strategy for GIOP prevention and treatment.

N^6^‐methyladenosine (m^6^A) RNA methylation is an emerging molecular mechanism that regulates gene expression at the post‐transcriptional level and represents the most prevalent epitranscriptomic modification in eukaryotic messenger RNA (mRNA).^10^, ^11^ This modification is dynamically deposited, removed and recognized by m^6^A methyltransferases (writers), demethylases (erasers) and m^6^A‐specific binding proteins (readers).^12^, ^13^, ^14^ YTH m^6^A RNA binding protein F1 (YTHDF1) is a crucial cytoplasmic m^6^A reader that promotes translation by interacting with initiation factors and ribosomes.^15^, ^16^, ^17^ Our previous research has shown that YTHDF1 is linked to osteogenic differentiation in human and mouse BMSCs, partly by enhancing Runx2 transcriptional activity via its downstream target Zfp839.^18^ Furthermore, RIP‐seq suggests that DNA damage‐regulated autophagy modulator 1 (DRAM1) is a potential YTHDF1 target, although their precise relationship and the role of YTHDF1 in GC‐induced osteogenic inhibition remain unclear.

DRAM1 is a highly conserved protein with significant homology across various organisms, primarily localized to key organelles such as lysosomes, early and late endosomes and the Golgi apparatus.^19^, ^20^ It not only participates in the regulation of autophagosome formation‐related complexes, such as ULK1 and ATG13, but also plays a crucial role in the fusion of autophagosomes with lysosomes. Additionally, DRAM1 can modulate autophagy by promoting lysosomal acidification and influencing lysosomal enzyme activity.^21^, ^22^, ^23^ Previous studies have reported elevated DRAM levels in osteoblasts of ovariectomized (OVX) rats, where it promotes autophagy and apoptosis.^24^ However, the effect of long‐term, high‐dose GCs on DRAM1 expression and its role in GC‐induced osteogenic inhibition have not been fully elucidated.

To the best of our knowledge, this is the first study to elucidate the role of YTHDF1 in regulating DRAM1‐dependent autophagy under GC exposure in BMSCs. We identify a previously unrecognized m^6^A‐dependent YTHDF1/DRAM1 regulatory axis that modulates autophagy and mediates GC‐induced osteogenic inhibition. This novel regulatory pathway not only expands our understanding of the epitranscriptomic mechanisms underlying bone metabolism but also identifies a promising molecular target for the treatment of GIOP.

## 2 MATERIALS AND METHODS

### 2.1 Cell culture and treatments

Human bone marrow mesenchymal stem cells (hBMSCs) were isolated from eight healthy male donors (age 20–30 years, mean age 26.5 years) undergoing intramedullary nailing for femoral fractures. Cell isolation and passaging were performed as previously described.^25^ Informed consent was obtained from all participants, and the study was approved by the ethics committee of Xinhua Hospital. The hBMSCs were used for experiments at passages 3 to 7.^18^ MC3T3‐E1 cells (CRL‐2593, ATCC) were used for experiments at passages below 20.^26^

hBMSCs and MC3T3‐E1 cells were cultured in α‐MEM (BL306A, Biosharp) supplemented with 10% fetal bovine serum (FSP500, ExCell) at 37°C in a humidified atmosphere containing 5% CO_2_. For osteogenic differentiation, cells were cultured in medium containing 4 mM β‐glycerophosphate (G9891, Sigma‐Aldrich) and 25 µg/mL ascorbic acid (A4403, Sigma‐Aldrich) until they reached 70% confluence.^21^, ^27^ Dexamethasone was then added for 2 weeks, with media changes every 2 days. Additionally, as per the protocol, cells were treated with 10^−5^ M RU486 (HY‐13683, MCE), 5 µg/mL SC79 (HY‐18749, MCE) for 48 h, 10 µM MK‐2206 (HY‐10358, MCE) for 48 h, 10 µM PF‐4708671 (HY‐15773, MCE) for 16 h, 100 nM Baf‐A1 (HY‐100558, MCE) for 1 h, or 100 nM rapamycin (HY‐10219, MCE) for 24 h as needed.

### 2.2 CCK‐8 analysis

Cell viability was assessed using a cell counting kit‐8 (CCK‐8) assay (C0039, Beyotime). hBMSCs and MC3T3‐E1 cells were seeded evenly in 96‐well plates and incubated overnight. The cells were then treated with various concentrations of dexamethasone for 48 h. After treatment, 10 µL of CCK‐8 solution was added to each well, and the plates were incubated for an additional 2 h. Absorbance was measured at 450 nm using a microplate reader.^28^

### 2.3 Cell cycle analysis

hBMSCs and MC3T3‐E1 cells were seeded evenly in six‐well plates and treated with α‐MEM containing 10^−5^ M dexamethasone for 48 h, while untreated cells served as the control. The cell cycle and apoptosis analysis kit (C1052, Beyotime) was used. Collected cells were washed with PBS, resuspended, fixed in 70% ethanol, stained with propidium iodide and then analyzed for cell cycle distribution by flow cytometry.^29^

### 2.4 ALP and Alizarin Red S staining

After inducing osteogenic differentiation (ALP staining for 1 week and Alizarin Red S (ARS) staining for 2 weeks), hBMSCs and MC3T3‐E1 cells were washed three times with PBS and fixed in 4% paraformaldehyde for 20 min. ALP staining was performed using the BCIP/NBT kit (C3206, Beyotime) for 30 min. ARS staining was conducted for 15 min using a solution prepared from Alizarin Red S powder (ST1078‐25g, Beyotime). After three PBS washes, stained cells in each well were photographed. Each staining experiment was repeated at least five times. The intensity of ALP and ARS staining was quantified using ImageJ software.

### 2.5 Western blotting

Total proteins were extracted from hBMSCs and MC3T3‐E1 cells using RIPA lysis buffer (P0013B, Beyotime) supplemented with protease and phosphatase inhibitors (P1045, Beyotime). The proteins were separated by SDS‐PAGE and transferred onto polyvinylidene difluoride (PVDF) membranes (IPVH00010, Millipore). Membranes were blocked with a protein‐free blocking buffer (PS108P, EpiZyme) for 15 min and incubated overnight at 4°C with primary antibodies. The primary antibodies used included YTHDF1 (17479‐1‐AP, Proteintech), DRAM1 (TA306390, Origene), RUNX2 (20700‐1‐AP, Proteintech), BMP2 (66383‐1‐Ig, Proteintech), OCN (ab309521, Abcam), LC3 (14600‐1‐AP, Proteintech), p62 (5114S, Cell Signaling Technology), p‐AKT (4060T, Cell Signaling Technology), AKT (4691T, Cell Signaling Technology), p‐mTOR (5536P, Cell Signaling Technology), mTOR (2983T, Cell Signaling Technology), p‐S6 (29223‐1‐AP, Proteintech), S6 (66886‐1‐Ig, Proteintech), p‐ULK1 (14202T, Cell Signaling Technology), ULK1 (8054T, Cell Signaling Technology), β‐catenin (8480T, Cell Signaling Technology), active β‐catenin (8814T, Cell Signaling Technology), and GAPDH (10494‐1‐AP, Proteintech). After three washes with TBST, membranes were incubated with HRP‐conjugated secondary antibodies for 1 h. Protein bands were visualized using the Ultrahypersensitive ECL kit (P0018AS, Beyotime).^30^

### 2.6 Quantitative real‐time PCR

Total RNA was extracted from cultured cells using TRIzol reagent (R0016, Beyotime) and reverse transcribed into cDNA using the PrimeScript RT Master Mix kit (RR036A, Takara). Subsequently, qRT‐PCR was performed using the SYBR Green Master Mix (11202ES08, Yeasen Biotechnology, Shanghai, China). Analysis was conducted using an ABI 7500 real‐time PCR system, following the manufacturer's instructions. Relative gene expression levels were normalized to glyceraldehyde 3‐phosphate dehydrogenase (GAPDH). Table S1 lists the primers used in the experiment.

### 2.7 Lentiviral infection, siRNA transfection, and plasmid transfection

MC3T3‐E1 cells were infected with Puro Lentivirus, PGMLV‐CMV‐Mouse_Ythdf1‐3×Flag‐PGK‐Puro, PGMLV‐EF1a‐EGFP‐H_LC3‐PGK‐Puro, or PGMLV‐EF1a‐TurboRFP‐EGFP‐H_LC3‐PGK‐Puro (Genomeditech, Shanghai, China) at approximately 30% confluence. Infection was performed in α‐MEM containing 5 µg/mL polybrene. After 24 h, the medium was replaced with fresh α‐MEM, and after 48 h, cells were selected with puromycin to obtain stably infected cells.

For siRNA transfection, MC3T3‐E1 cells at approximately 70% confluence were transiently transfected with si‐NC, si‐Ythdf1, or si‐Dram1 (Genomeditech) using Rfect siRNA/miRNA Transfection Reagent (11012, Baidai Biotechnology, Changzhou, China).^12^ Gene silencing efficiency was assessed 72 h post‐transfection.

For plasmid transfection, constructs—pcDNA3.1, pcDNA3.1‐Ythdf1 (GenePharma, Shanghai, China), PIVX‐PGK‐Puro, PGMLV‐CMV‐Mouse_Dram1‐3×Flag‐PGK‐Puro, PGL3‐CMV‐LUC‐Mouse_Dram1 3′UTR WT, PGL3‐CMV‐LUC‐Mouse_Dram1 3′UTR MUT or pGMR‐TK (Genomeditech)—were mixed with serum‐free medium and P3000 reagent. This mixture was then combined with Lipofectamine 3000 (L3000001, Invitrogen, Thermo Fisher Scientific) prepared in an equal volume of serum‐free medium, incubated and subsequently added to the cells. Cells were cultured for 72 h before further experiments.^18^

### 2.8 Methylated RNA immunoprecipitation

The MeRIP assay was performed using the GenSeq m^6^A MeRIP kit (GenSeq Inc.) according to the manufacturer's instructions. RNA was diluted to 1 µg/µL and fragmented by incubation with Fragmentation Buffer at 70°C for 6 min, and the reaction was stopped with Stop Buffer. The fragmented RNA was precipitated with PC buffer, PC enhancer and anhydrous ethanol at −20°C or −80°C, followed by centrifugation to collect the pellet and washing with 75% ethanol. After dissolving the RNA, its size and concentration were assessed using a NanoDrop spectrophotometer. A portion was reserved as an input control, while the remainder was used for immunoprecipitation. Magnetic beads were washed and incubated with an anti‐m^6^A antibody for 1 h, followed by incubation of the antibody–bound beads with RNA at 4°C for 1 h. After washing, the beads were resuspended in RLT buffer, and the supernatant was transferred to a new tube. The supernatant was then incubated with pre‐washed MS beads and treated with anhydrous ethanol. After washing with 75% ethanol and drying, RNA was eluted with nuclease‐free water and used for subsequent analyses.

### 2.9 RNA immunoprecipitation

RNA immunoprecipitation (RIP) assays were performed using the Magna RIP Kit (17‐700, Millipore) to assess the direct association between YTHDF1 and DRAM1 mRNA. Samples were lysed in RIP lysis buffer supplemented with RNase inhibitors and a protease inhibitor cocktail. Protein A/G magnetic beads were pre‐incubated with either anti‐YTHDF1 antibody or control IgG, and then mixed with the lysates and rotated overnight at 4°C. Proteinase K was used to digest the beads following washing with RIP washing buffer. The RNA from both the input and immunoprecipitated samples was isolated using 100% ethanol and phenol/chloroform/isoamyl alcohol (125:24:1). The RNA was reverse transcribed into cDNA and quantified by RT‐qPCR.^31^

### 2.10 Bioinformatics analysis

The RIP‐seq scatter plot was generated based on the uploaded data link (https://share.weiyun.com/xcB3waFn). RPISeq (http://pridb.gdcb.iastate.edu/RPISeq/index.html) was applied to predict potential RNAprotein interactions. The distribution of m^6^A modification motifs in the target mRNA was predicted using the m^6^A site predictor SRAMP (http://www.cuilab.cn/sramp).

### 2.11 Animals and grouping

Ythdf1 CRISPR‐Cas9 knockout (Ythdf1 KO) mice were generously provided by the School of Life Science and Technology, ShanghaiTech University, and were constructed as previously described.^32^, ^33^ Genotyping of offspring was confirmed via PCR analysis of tail DNA. All procedures complied with NIH guidelines for the care and use of laboratory animals. Mice were housed under standard conditions (21 ± 2°C, 60% ± 10% humidity) with five mice per cage. Ten‐week‐old male WT and Ythdf1 KO mice were used (*n* = 5 per group).

WT male C57BL/6 mice (*n* = 5) were used for RIP‐qPCR assays. Bilateral femurs were collected from each mouse, and tissue lysates were subjected to immunoprecipitation with either an anti‐YTHDF1 antibody or IgG as a negative control. Additionally, femurs from WT male C57BL/6 mice in the control group (*n* = 5) and mice treated with intraperitoneal dexamethasone for 8 weeks (*n* = 5) were used for MeRIP‐qPCR assays to evaluate the m^6^A methylation levels of Dram1 mRNA.

WT male C57BL/6 mice were randomly assigned to three groups (*n* = 5 per group): Control, Dexamethasone, and Dexamethasone + Resveratrol, and received intraperitoneal injections accordingly. Dexamethasone (25 mg/kg body weight) (HY‐14648, MCE) was administered via intraperitoneal injection for 8 weeks, with resveratrol (40 mg/kg body weight) (HY‐16561, MCE) co‐administered in the combination group.^34^ Mice were euthanized, and femurs were collected for further analysis. Group allocation was performed using a computer‐generated random number table. All animal handling and data analysis were performed in a blinded manner with respect to group assignments.

### 2.12 Tail‐biopsy genotyping

For genotyping, scissors were sterilized by autoclaving and alcohol disinfection. A small tail section was collected from each mouse and placed into a microcentrifuge tube.

The One Step Mouse Genotyping Kit (PD101‐01, Vazyme) was used. Lysis buffer was added to the tube and vortexed to mix, followed by incubation at 55°C for 20 min. The temperature was increased to 95°C for 5 min to inactivate proteinase K. The samples were vortexed again and centrifuged at 12 000 rpm for 5 min. A total of 100 µL of the supernatant was transferred to a new tube, avoiding the pellet.

PCR was set up according to the genotyping primer kit instructions. TAE buffer was prepared by mixing 20 mL of TAE (ST716, Beyotime) with 1 L of distilled water. One hundred millilitres was poured into a flask, agarose powder was added to make a 1.5% gel, and the mixture was heated until boiling. The solution was cooled to 50°C, 10 µL of Gel Red stain (diluted 1:10 000) (41003, Biotium) was added, mixed and poured into a mould to solidify. Once solid, the gel was placed in an electrophoresis tank filled with TAE buffer.

The comb was removed, 2 µL of each sample was loaded, and agarose gel electrophoresis was performed at 100 V for 20 min. DNA bands were analyzed using a gel imaging system (GELDOC XR+GEL, BioRad). Table S2 lists the primers used in the experiment.

### 2.13 Immunofluorescence and confocal microscopy

For immunofluorescence staining, decalcified paraffin‐embedded mouse femur sections or paraformaldehyde‐fixed cells were used. Sections or coverslips were permeabilized with Triton X‐100 and blocked with BSA. They were then incubated overnight at 4°C with primary antibodies against YTHDF1, DRAM1 and active β‐catenin. The primary antibodies used included YTHDF1 (17479‐1‐AP, Proteintech), DRAM1 (TA306390, Origene), active β‐catenin (ab246504, Abcam) and LAMP2 (66301‐1‐Ig, Proteintech). Afterwards, fluorescently labelled secondary antibodies were applied, and cell nuclei were counterstained with DAPI. Staining was examined using an Olympus fluorescence microscope (Olympus BX51).

For confocal microscopy, MC3T3‐E1 cells were infected with fluorescent PGMLV‐EF1a‐EGFP‐H_LC3‐PGK‐Puro or PGMLV‐EF1a‐TurboRFP‐EGFP‐H_LC3‐PGK‐Puro, seeded onto coverslips and treated with drugs or siRNAs. After fixing with 4% paraformaldehyde and permeabilizing with 0.2% Triton X‐100, cells were stained with DAPI to visualize nuclei. Fluorescence was observed using a confocal microscope.

### 2.14 Luciferase reporter assay

The assay was performed using the dual luciferase reporter gene assay kit (11402ES60, Yeasen Biotechnology). The cell culture medium was removed from the 24‐well plates, and 200 µL of cell lysis buffer was added to each well. The plates were incubated on ice for 5 min to allow for complete cell lysis. Subsequently, 20 µL of the lysate from each well was transferred to a white opaque 96‐well plate, and each sample was assayed in triplicate. Firefly luciferase reaction buffer and Renilla luciferase reaction buffer were prepared according to the manufacturer's instructions. A microplate luminometer (LB960, Berthold) was used to measure luminescence.

### 2.15 RNA pull‐down

RNA pull‐down assays were performed using the Pierce Magnetic RNA‐protein pull‐down kit (20164, Thermo Fisher Scientific). Biotin‐labelled wild‐type and m^6^A‐mutated Dram1 RNA transcripts were synthesized by in vitro transcription and purified. The RNA probes were incubated with protein lysates from MC3T3‐E1 cells. RNA–protein complexes were pulled down, eluted, denatured and analyzed by SDS‐PAGE followed by western blotting. YTHDF1 protein levels were detected to assess RNA–protein interactions.

### 2.16 Ribosome nascent–chain complex‐mRNA isolation

Cells were pretreated with cycloheximide (100 µg/mL) at 37°C for 15 min, followed by lysis on ice for 30 min in lysis buffer. The lysis buffer was prepared by mixing 1% Triton X‐100 with ribosome buffer containing 20 mM HEPES‐KOH (pH 7.4), 15 mM MgCl_2_, 200 mM KCl, 100 µg/mL cycloheximide and 2 mM dithiothreitol. The cell lysates were centrifuged at 16 200×*g* for 10 min at 4°C. Ten percent of the lysate was saved as the input control, and the remaining lysate was layered onto 30% sucrose buffer, followed by ultracentrifugation at 174 900×*g* for 5 h at 4°C. The resulting pellets were harvested, and RNA was extracted using TRIzol. Total RNA from the input control and ribosome nascent–chain complex (RNC) samples was subjected to RT‐qPCR for further analysis.^17^, ^35^

### 2.17 Transmission electron microscopy

Cell samples were centrifuged to form pellets of approximately pea size and fixed in electron microscopy fixative at 4°C for 4 h. After fixation, samples were washed three times with PBS (15 min each) and post‐fixed in 1% osmium tetroxide at room temperature for 2 h, followed by three additional PBS washes. Samples were then dehydrated in a graded ethanol series (50% to 100%, 15 min per concentration) and infiltrated overnight in a 1:1 mixture of acetone and 812 embedding medium, followed by pure 812 embedding medium. The samples were embedded at 60°C for 48 h, and ultrathin sections (80 nm) were cut and stained with 2% uranyl acetate and lead citrate (15 min each). Sections were air‐dried overnight at room temperature and examined under a transmission electron microscope for imaging and analysis.

### 2.18 Micro‐computed tomography scanning and quantitative analysis

Femurs were fixed in 4% paraformaldehyde and scanned using a micro‐CT scanner (SkyScan 1275, Bruker, Belgium) with an 8 µm resolution. The region of interest (ROI) was set to the trabecular bone 1.6 mm from the distal femur growth plate. Both two‐dimensional and three‐dimensional images were obtained to assess microstructural parameters, including bone mineral density (BMD), bone volume/total volume (BV/TV), trabecular number (Tb.N), trabecular pattern factor (Tb.Pf), trabecular separation (Tb.Sp), trabecular thickness (Tb.Th), mean total cross‐sectional bone area (B.Ar), mean total cross‐sectional tissue area (T.Ar) and cross‐sectional thickness (Cs.Th).^36^

### 2.19 Calcein double‐labelling analysis and von Kossa staining

Mice received intraperitoneal injections of calcein (10 mg/kg) on days 8 and 3 before euthanasia. After euthanasia, femurs were collected, fixed in 4% paraformaldehyde and embedded in methyl methacrylate. Sections (8 µm thick) were prepared using the EXAKT cutting and grinding system and analyzed for calcein double‐labelling under a fluorescence microscope.

For von Kossa (VK) staining, hard tissue sections were incubated with 5% silver nitrate solution under UV light for 1 h, then with 5% sodium thiosulfate for 2–3 min. After thoroughly rinsing with distilled water, the sections were dehydrated in absolute ethanol, cleared in xylene and mounted with DPX.

### 2.20 Hematoxylin–eosin and immunohistochemical staining

The femurs fixed in 4% paraformaldehyde were decalcified in 10% EDTA (pH 7.0) for 21 days, embedded in paraffin and sectioned. For hematoxylin‐eosin staining, sections were stained with hematoxylin for 5 min and eosin for 2 min. For immunohistochemistry, sections (8 µm thick) were incubated overnight with primary antibodies at 4°C. The primary antibodies used included DRAM1 (TA306390, Origene) and COL1A1 (72026, Cell Signaling Technology). Immunoreactivity was detected using a horseradish peroxidase‐streptavidin system (Dako, Glostrup, Sweden), with nuclei counterstained with hematoxylin.

### 2.21 TRAP staining

Sections were deparaffinized, rehydrated and preincubated in 0.1 M acetate buffer. The TRAP staining solution, containing sodium acetate buffer and naphthol AS‐BI phosphate, was applied and incubated at room temperature for 50 min. Following staining, sections were washed with acetate buffer, rinsed with distilled water and air‐dried. Subsequently, hematoxylin was used to counterstain the cell nuclei. TRAP‐positive cells, indicative of osteoclast activity, were visualized as red or pink under a light microscope.

### 2.22 Statistical analysis

All experiments were performed with a minimum of three independent replicates. Data are presented as the mean ± standard deviation. Comparisons between two groups were conducted using a two‐tailed Student's *t*‐test, while comparisons among multiple groups were performed using one‐way analysis of variance (ANOVA) followed by Tukey's test.^37^ A *p*‐value <.05 was considered statistically significant. Statistical significance is indicated as ***p < .001, **p < .01 and *p < .05.

## 3 RESULTS

### 3.1 High‐dose dexamethasone stimulation downregulates YTHDF1 and DRAM1 expression in hBMSCs and MC3T3‐E1 cells

**FIGURE 1:**
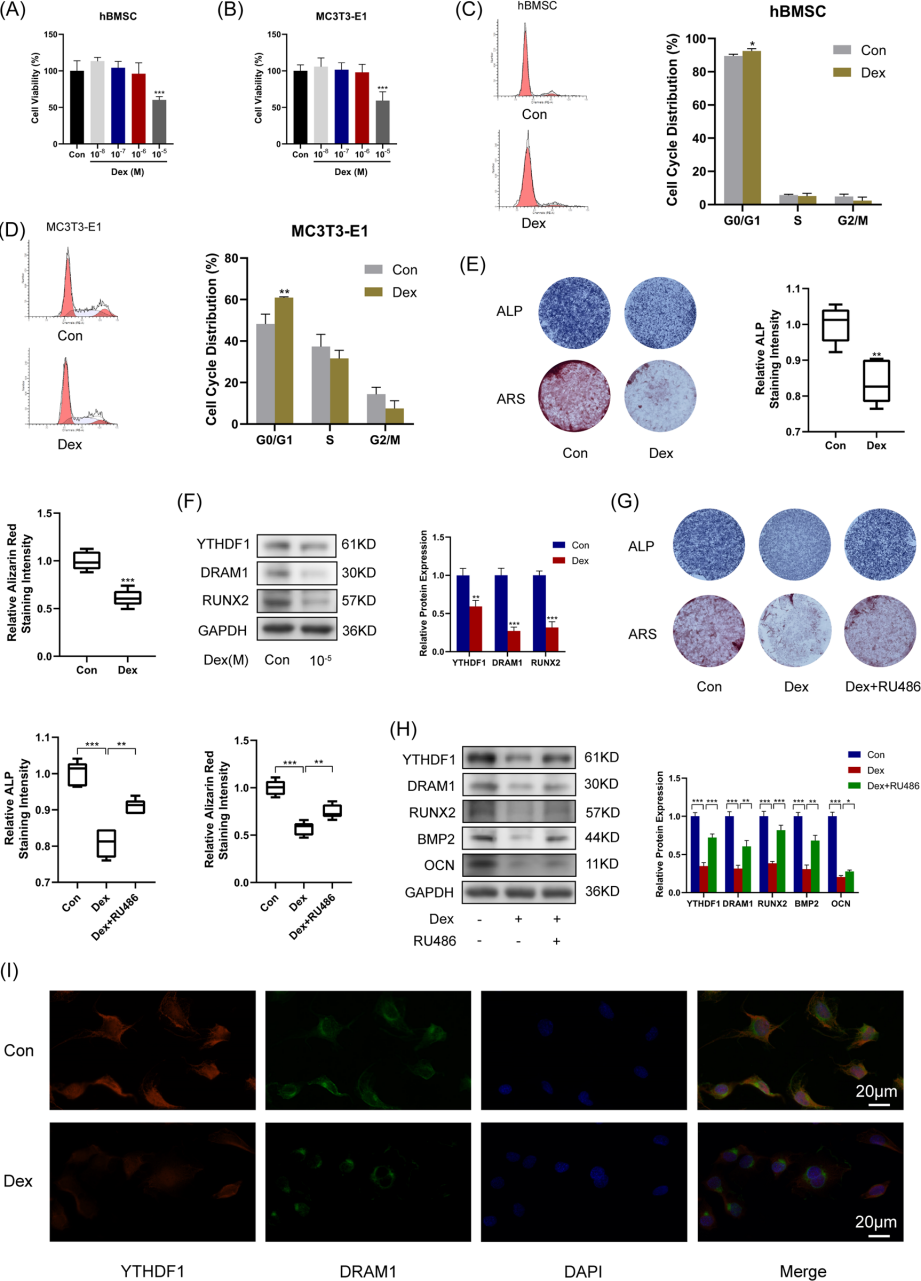
High‐dose dexamethasone reduces cell viability and osteogenesis and downregulates YTHDF1 and DRAM1. (A, B) Significant reduction in cell viability in hBMSCs and MC3T3‐E1 cells under 10^−5 ^M dexamethasone. (C, D) Increased G0/G1 phase proportion in hBMSCs and MC3T3‐E1 cells with 10^−5^ M dexamethasone. (E) ALP and ARS assays revealed inhibited osteogenesis in hBMSCs after high‐dose dexamethasone treatment. (F) Western blot analysis showed reduced expression of YTHDF1 and DRAM1 in dexamethasone‐treated hBMSCs. (G) RU486 reversed dexamethasone‐induced osteogenic inhibition in ALP and ARS assays in MC3T3‐E1 cells. (H) RU486 increased YTHDF1, DRAM1 and osteogenic protein expression levels (e.g., OCN) in MC3T3‐E1 cells compared with dexamethasone alone. (I) Immunofluorescence showed that high‐dose dexamethasone treatment led to a reduction in YTHDF1 and DRAM1 expression in MC3T3‐E1 cells. Con, control; Dex, dexamethasone; RU486, mifepristone.

To examine the inhibitory effect of dexamethasone on hBMSCs and MC3T3‐E1 cell viability, we tested concentrations ranging from 10^−8^ to 10^−5^ M. Cell viability was significantly decreased at 10^−5^ M compared with the control (Figure 1A,B). Flow cytometry revealed an increased proportion of cells in the G0/G1 phase under 10^−5^ M dexamethasone, with minimal changes in the S and G2/M phases, indicating G0/G1 phase arrest and reduced viability (Figure 1C,D).

To assess the impact of high‐dose (10^−5^ M) dexamethasone on osteogenesis, ALP and ARS assays showed marked inhibition of osteogenic capacity in hBMSCs (Figure 1E). Western blot analysis confirmed reduced expression of osteogenic markers, such as RUNX2, in dexamethasone‐treated cells. Although YTHDF1 and DRAM1 have been implicated in osteogenesis, their precise roles and responses to long‐term high‐dose glucocorticoid treatment have not been fully explored. Our results showed that both YTHDF1 and DRAM1 were significantly downregulated under high‐dose dexamethasone stimulation (Figure 1F).

Further investigations in hBMSCs and MC3T3‐E1 cells revealed that expression levels of YTHDF1 and DRAM1, along with osteogenic genes such as RUNX2, were upregulated at dexamethasone concentrations below 10^−7^ M, indicative of enhanced osteogenic differentiation. In contrast, higher concentrations led to downregulation of YTHDF1, DRAM1 and RUNX2, suggesting inhibited osteogenesis (Figure S1A,B). In MC3T3‐E1 cells, RT‐qPCR analysis showed that treatment with 10^−5^ M dexamethasone significantly reduced Ythdf1 mRNA expression, whereas Ythdf2, Ythdf3, Ythdc1 and Ythdc2, encoding proteins with similar YTH domains, remained largely unchanged (Figure S1C). Notably, Dram1 mRNA expression was not significantly altered under the same conditions, suggesting that dexamethasone may not directly affect Dram1 mRNA stability but could modulate its protein expression through post‐transcriptional regulation (Figure S1D).

To confirm the inhibitory effect of dexamethasone on osteogenesis, we treated cells with RU486 (mifepristone), a dexamethasone antagonist, at 10^−5^ M. ALP and ARS assays demonstrated that RU486 reversed the osteogenic inhibition (Figure 1G). Western blotting showed that cells treated with both dexamethasone and RU486 exhibited significantly higher levels of YTHDF1, DRAM1 and osteogenic markers such as OCN compared with dexamethasone alone (Figure 1H). Immunofluorescence confirmed decreased YTHDF1 and DRAM1 expression under high‐dose dexamethasone (Figure 1I), highlighting potential regulatory changes. However, whether YTHDF1 mediates dexamethasone‐induced osteogenic inhibition via DRAM1 remains unclear.

### 3.2 YTHDF1 binds to m^6^A‐modified DRAM1 mRNA and promotes its translation

**FIGURE 2:**
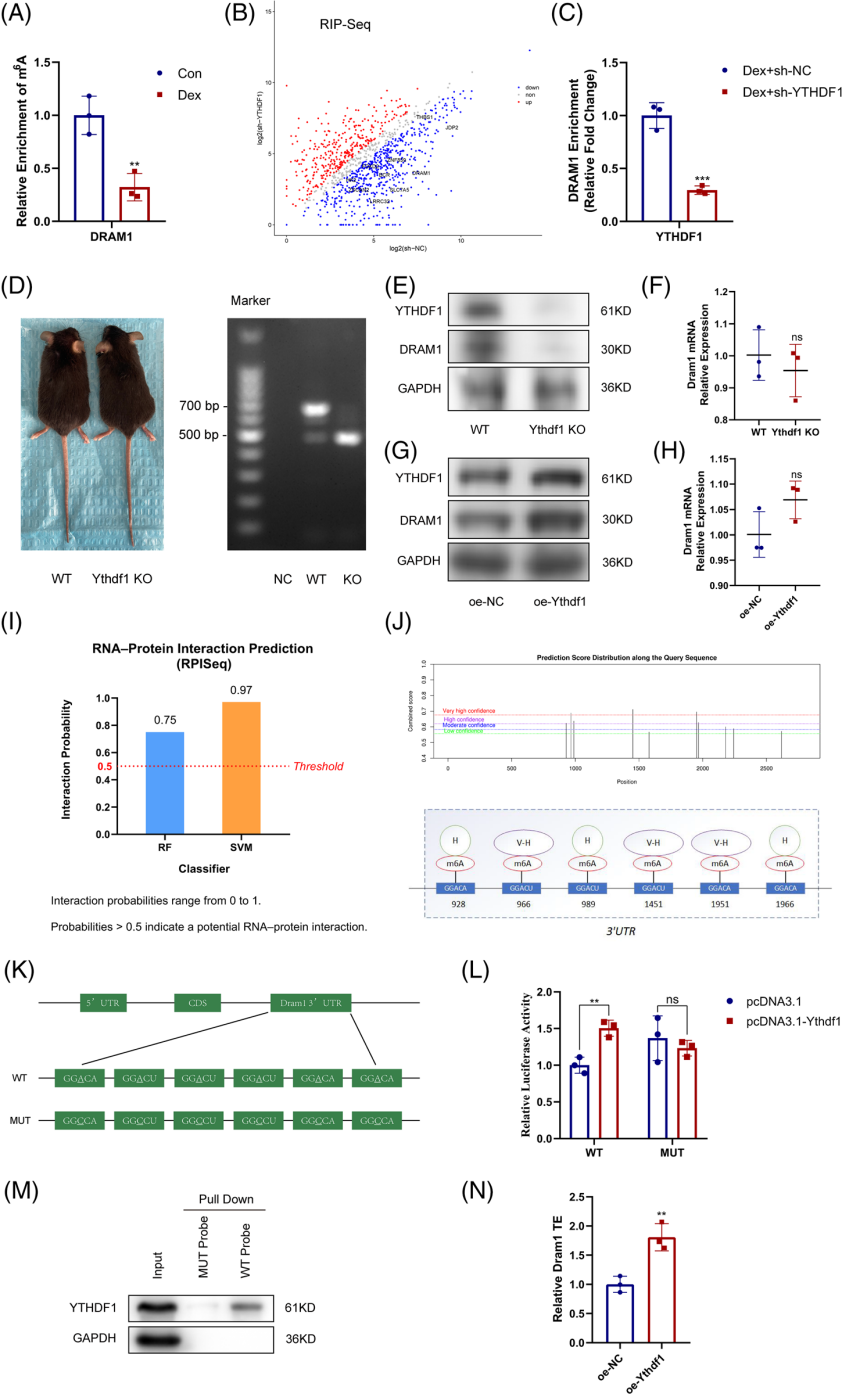
YTHDF1 binds to and promotes translation of m^6^A‐modified DRAM1 mRNA. (A) MeRIP‐qPCR showed decreased m^6^A levels of DRAM1 in hBMSCs under high‐dose dexamethasone compared with untreated cells. (B) RIP‐seq revealed reduced binding between DRAM1 mRNA and YTHDF1 in YTHDF1 knockdown hBMSCs. (C) RIP‐qPCR demonstrated reduced binding of DRAM1 mRNA to YTHDF1 in hBMSCs treated with sh‐YTHDF1 under dexamethasone. (D) Tail genotyping distinguished Ythdf1 KO mice from WT mice. (E, F) Western blot showed reduced DRAM1 protein in Ythdf1 KO mouse femurs, while qPCR showed no significant difference in Dram1 mRNA levels between the groups. (G, H) In MC3T3‐E1 cells overexpressing Ythdf1, Western blot showed increased DRAM1 protein, with qPCR showing no difference in mRNA levels between groups. (I) RPISeq predicted the interaction probability between YTHDF1 and Dram1 mRNA. (J) SRAMP predicted three high‐confidence and three very high‐confidence m^6^A sites within the 3′UTR of the mouse Dram1 transcript. (K) Construction of a reporter gene plasmid with all six m^6^A sites mutated. (L) Luciferase assays in HEK‐293T cells showed increased luciferase activity in cells co‐transfected with the Ythdf1 overexpression plasmid and Dram1‐WT plasmid, but no change in cells transfected with the Dram1‐MUT plasmid. (M) RNA pull‐down showed greater YTHDF1 enrichment with wild‐type Dram1 probes than with m^6^A‐mutated ones in MC3T3‐E1 cells. (N) RNC‐qPCR showed enhanced Dram1 mRNA translation in Ythdf1‐overexpressing MC3T3‐E1 cells compared with the oe‐NC group. Con, control; Dex, dexamethasone; KO, knockout; m^6^A, N^6^‐methyladenosine; MUT, mutant; RIP, RNA immunoprecipitation; TE, translation efficiency; WT, wild type.

To elucidate how YTHDF1, an m^6^A ‘reader’, regulates DRAM1 in the context of GIOP, we first performed MeRIP‐qPCR to assess the m^6^A modification status of DRAM1 mRNA. The results showed that hBMSCs treated with 10^−5^ M dexamethasone for 2 weeks exhibited significantly reduced m^6^A enrichment on DRAM1 transcripts compared with untreated controls, suggesting that glucocorticoid exposure decreases m^6^A methylation of DRAM1 (Figure 2A). To further evaluate whether YTHDF1 protein directly binds to DRAM1 mRNA, we re‐analyzed RIP‐seq data from our previously published study, in which RIP was performed using an anti‐YTHDF1 antibody in hBMSCs transfected with either sh‐NC or sh‐YTHDF1.^18^ In the YTHDF1 knockdown group, DRAM1 enrichment was markedly reduced, showing an approximately 19.25‐fold decrease in binding peak intensity (Figure 2B). Similar results were observed under dexamethasone treatment using previously constructed shRNAs (Figure 2C). These findings suggest that YTHDF1 may regulate DRAM1 through an m^6^A‐dependent mechanism during dexamethasone‐induced osteogenic inhibition.

To confirm the YTHDF1‐Dram1 interaction in vivo, we performed RIP‐qPCR using RNA extracted from femurs of wild‐type (WT) mice. Dram1 mRNA was significantly enriched by the anti‐YTHDF1 antibody compared with the IgG control, indicating binding between YTHDF1 and Dram1 mRNA in bone tissue (Figure S2A). Additionally, MeRIP‐qPCR analysis revealed a marked reduction in m^6^A enrichment on Dram1 transcripts in femurs from mice subjected to long‐term intraperitoneal dexamethasone injection, compared with controls (Figure S2B). These findings suggest that glucocorticoid exposure decreases m^6^A methylation of Dram1 in vivo, potentially impairing its recognition by YTHDF1. Tail genotyping was performed to distinguish Ythdf1 knockout (KO) mice from WT littermates (Figure 2D). Western blot analysis revealed significantly reduced DRAM1 protein expression in femurs from Ythdf1 KO mice compared with WT mice (Figure 2E), consistent with immunofluorescence results (Figure S2C). However, qPCR analysis showed no significant difference in Dram1 mRNA levels between Ythdf1 KO and WT mice (Figure 2F). Similarly, in MC3T3‐E1 cells overexpressing Ythdf1 via lentivirus infection (Figure S2D,E), western blot analysis showed increased DRAM1 protein levels (Figure 2G), whereas qPCR revealed no significant change in Dram1 mRNA levels (Figure 2H). These results suggest YTHDF1 may regulate Dram1 translation by binding to its mRNA. This potential interaction was also predicted by the RPISeq tool (Figure 2I).

Using the SRAMP tool, we identified three high‐confidence m^6^A sites (loci 928, 989 and 1966) and three very high‐confidence sites (loci 966, 1451 and 1951) within the 3′UTR of Dram1 mRNA (Figure 2J), with no predicted sites in the CDS or 5′UTR. We then generated a reporter plasmid containing mutations at all six m^6^A sites (Dram1‐MUT) (Figure 2K) and constructed a Ythdf1 overexpression plasmid (Figure S2F). Luciferase assays in HEK‐293T cells showed that Ythdf1 overexpression significantly increased luciferase activity in cells transfected with the wild‐type Dram1 plasmid (Dram1‐WT) containing intact m^6^A sites. In contrast, no significant change was observed in cells transfected with the mutated Dram1 plasmid (Dram1‐MUT) (Figure 2L). RNA pull‐down assays using biotin‐labeled Dram1‐WT and m^6^A site‐mutated Dram1‐MUT probes in MC3T3‐E1 cells demonstrated that YTHDF1 protein was significantly more enriched by the WT probe compared with the MUT probe, indicating that the binding of YTHDF1 to Dram1 mRNA is m^6^A‐dependent (Figure 2M). To further evaluate the effect of YTHDF1 on Dram1 translation, we performed RNC‐qPCR in MC3T3‐E1 cells. The results showed that overexpression of Ythdf1 significantly enhanced the translational efficiency of Dram1 mRNA compared with the oe‐NC group (Figure 2N). Collectively, these findings indicate that YTHDF1 binds to m^6^A‐modified Dram1 mRNA and promotes its translation.

### 3.3 YTHDF1 influences osteogenic potential via DRAM1 in dexamethasone‐exposed MC3T3‐E1 cells

**FIGURE 3:**
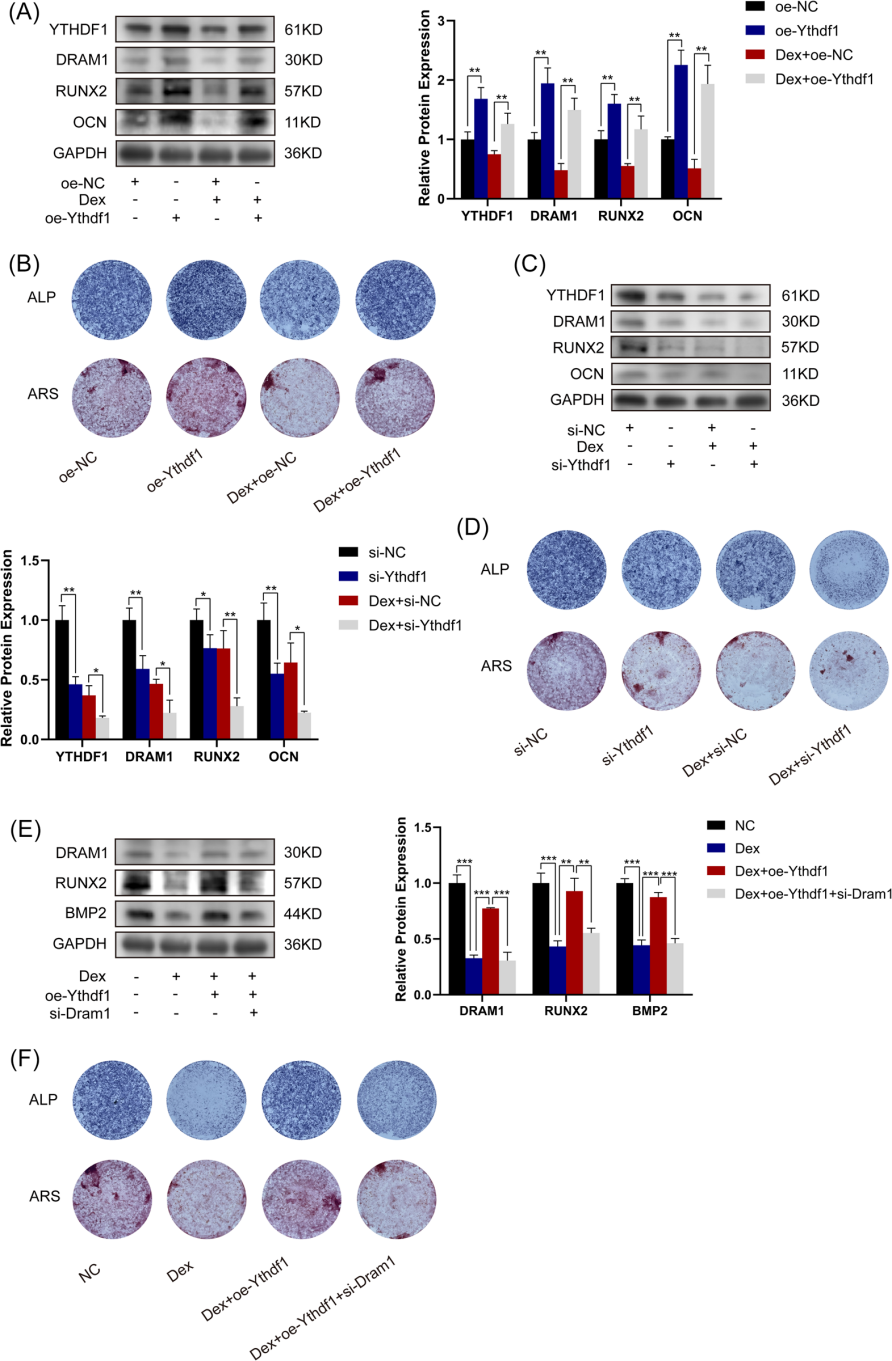
YTHDF1 regulates osteogenic potential through DRAM1 in dexamethasone‐exposed MC3T3‐E1 cells. (A) Western blot analysis showed increased DRAM1, RUNX2 and OCN levels in MC3T3‐E1 cells overexpressing Ythdf1. (B) ALP and ARS staining revealed enhanced osteogenic function in Ythdf1‐overexpressing cells under dexamethasone treatment. (C) siRNA‐mediated Ythdf1 knockdown reduced DRAM1, RUNX2 and OCN expression in dexamethasone‐treated cells. (D) ALP and ARS staining showed decreased osteogenic capacity in Ythdf1‐knockdown cells. (E) Co‐transfection of si‐Dram1 and infection with a Ythdf1 overexpression lentiviral vector reduced RUNX2 and BMP2 levels compared with Ythdf1 overexpression alone. (F) ALP and ARS staining demonstrated reduced osteogenic potential in MC3T3‐E1 cells co‐transfected with si‐Dram1. Dex, dexamethasone.

To explore the role of Ythdf1 in osteogenesis under long‐term high‐dose dexamethasone treatment, we performed loss‐ and gain‐of‐function experiments in MC3T3‐E1 cells. Western blot analysis revealed that Ythdf1 overexpression increased the protein levels of DRAM1, RUNX2 and OCN (Figure 3A). ALP and ARS staining further confirmed that Ythdf1 overexpression enhanced osteogenic capacity in these cells (Figure 3B).

We further investigated the role of Ythdf1 by using siRNAs to inhibit its expression. Among the three siRNAs tested, si‐Ythdf1‐3 was the most effective and was used in subsequent experiments (Figure S3A,B). Moreover, Dram1 mRNA expression showed no significant difference in cells transfected with si‐Ythdf1‐3 compared with the si‐NC group (Figure S3C). In dexamethasone‐treated cells transfected with si‐Ythdf1, DRAM1 protein expression and osteogenic markers (RUNX2 and OCN) were significantly reduced (Figure 3C). ALP and ARS staining further corroborated these inhibitory effects (Figure 3D), suggesting that Ythdf1 knockdown exacerbates dexamethasone‐induced osteogenic suppression.

To confirm whether Ythdf1 regulates osteogenic inhibition through Dram1, we co‐transfected MC3T3‐E1 cells overexpressing Ythdf1 with siRNA targeting Dram1. Among the three siRNAs, si‐Dram1‐3 was the most effective (Figure S3D,E). Western blot analysis showed that dexamethasone‐treated cells overexpressing Ythdf1 exhibited higher levels of osteogenic markers (RUNX2 and BMP2) compared with those co‐transfected with si‐Dram1 (Figure 3E). ALP and ARS staining showed reduced osteogenic capacity in cells with si‐Dram1 (Figure 3F). These results indicate that YTHDF1 modulates osteogenic potential in dexamethasone‐treated MC3T3‐E1 cells through regulation of DRAM1.

### 3.4 DRAM1 regulates autophagic activity via the AKT/rpS6 pathway and related signalling molecules

**FIGURE 4:**
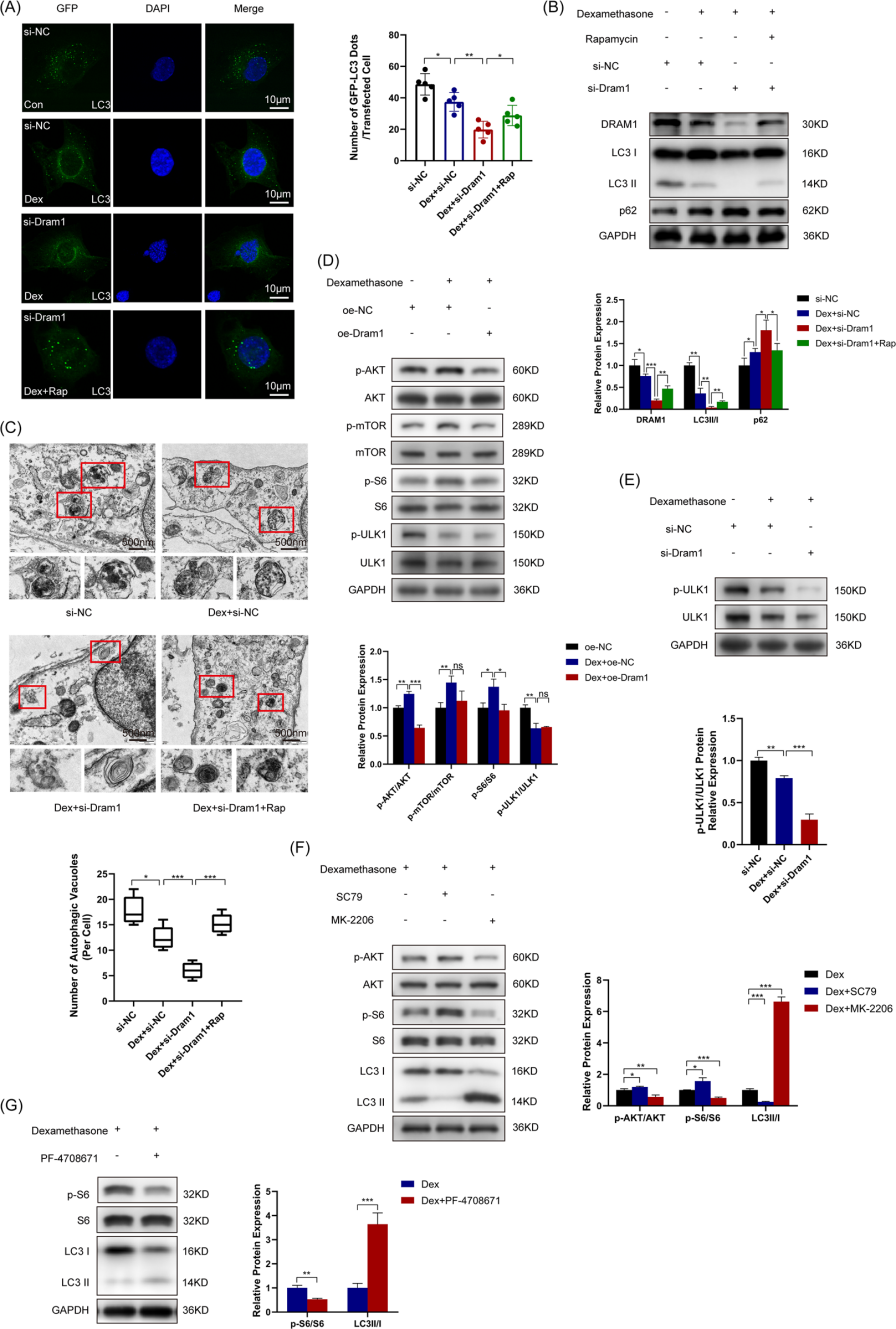
DRAM1 modulates autophagy via the AKT/rpS6 pathway in MC3T3‐E1 cells. (A) GFP‐LC3 staining showed fewer GFP‐LC3 dots in dexamethasone‐treated MC3T3‐E1 cells with si‐Dram1, indicating reduced autophagosome formation; rapamycin treatment increased GFP‐LC3 dots. (B) Western blot analysis demonstrated decreased LC3‐I to LC3‐II conversion and increased p62 levels in si‐Dram1 cells, which were reversed by rapamycin. (C) TEM images revealed fewer autophagic structures in si‐Dram1‐transfected cells, with rapamycin increasing these structures. (D) Western blot results showed lower p‐AKT and p‐S6 levels in Dram1‐overexpressing cells compared with dexamethasone‐only controls. (E) Reduced p‐ULK1 levels in si‐Dram1 cells, with no change in Dram1‐overexpressing cells. (F) SC79 treatment increased p‐S6 levels and decreased the LC3‐II/I ratio, whereas MK‐2206 exhibited the opposite effect. (G) PF‐4708671 treatment increased the LC3‐II/I ratio through inhibition of S6K1. Con, control; Dex, dexamethasone; Rap, rapamycin.

Previous studies have shown that DRAM1 influences cellular autophagic activity.^23^, ^38^ We infected MC3T3‐E1 cells with a lentivirus carrying GFP‐LC3 and then transfected them with si‐NC or si‐Dram1. In dexamethasone‐treated cells transfected with si‐Dram1, GFP‐LC3 dots were significantly reduced, indicating decreased autophagosome formation. Conversely, cells treated with rapamycin showed increased GFP‐LC3 dots, indicating enhanced autophagosome formation (Figure 4A). Molecular analysis revealed that Dram1 knockdown inhibited the conversion of LC3‐I to LC3‐II and increased p62 levels, while rapamycin reversed these effects (Figure 4B). Transmission electron microscopy (TEM) images showed fewer autophagic structures, such as autophagosomes and autolysosomes, in cells treated with dexamethasone and si‐Dram1 compared with controls, indicating a combined inhibitory effect on autophagy. Rapamycin treatment increased these structures, suggesting enhanced autophagic activity (Figure 4C).

To investigate the signalling pathways involved in DRAM1‐induced autophagy, we modulated Dram1 expression and examined relevant markers. Previous research highlighted the role of ribosomal protein S6 (rpS6) phosphorylation in inhibiting autophagic proteolysis in Wistar rat liver cells.^39^ This was further validated in HEK‐293T cells.^21^ We introduced a Dram1 overexpression plasmid into MC3T3‐E1 cells (Figure S4A,B). Western blot analysis showed that Dram1 overexpression in dexamethasone‐treated cells significantly reduced p‐AKT and p‐S6 levels compared with dexamethasone alone (Figure 4D), suggesting that DRAM1‐induced autophagy involves inhibition of rpS6 phosphorylation. Additionally, p‐ULK1 levels were reduced in dexamethasone‐treated cells transfected with si‐Dram1, but no significant change was observed in Dram1‐overexpressing cells (Figure 4D,E). To further investigate the role of the AKT pathway, cells were treated with the AKT activator SC79 or the AKT inhibitor MK‐2206. Cells treated with dexamethasone and SC79 exhibited increased p‐S6 protein expression and a decreased LC3‐II/I ratio compared with dexamethasone alone, whereas cells treated with MK‐2206 showed the opposite trend (Figure 4F). Treatment with the p70 S6 kinase inhibitor PF‐4708671, which prevents S6K1‐mediated phosphorylation of the S6 protein, significantly increased the LC3‐II/I ratio compared with the control group (Figure 4G). Taken together, these results indicate that DRAM1 regulates autophagic activity in MC3T3‐E1 cells via the AKT/rpS6 pathway and related signalling molecules.

### 3.5 DRAM1 regulates osteogenic differentiation by modulating autophagic flux

**FIGURE 5:**
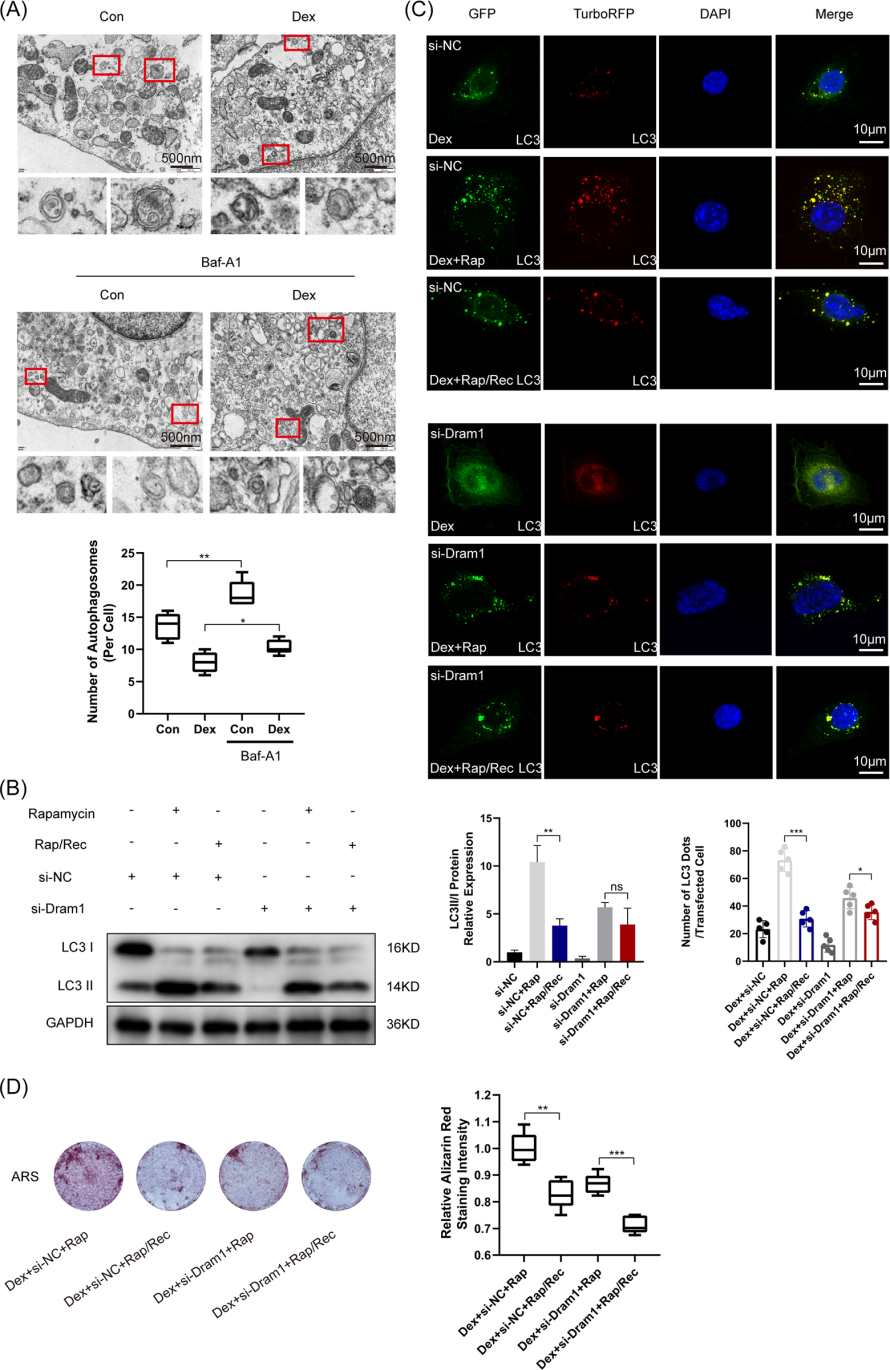
DRAM1 regulates autophagic flux in MC3T3‐E1 cells. (A) TEM analysis showed a reduced number of autophagosomes in dexamethasone‐treated cells compared with the control. Baf‐A1 increased autophagosome accumulation in both groups, with a more pronounced effect in the control group. (B) Western blot revealed that si‐Dram1 cells maintained elevated LC3‐II levels 8 h after rapamycin removal, unlike si‐NC control cells, where LC3‐II levels returned to near baseline. (C) GFP‐TurboRFP‐LC3 dual fluorescence microscopy showed impaired autophagosome clearance in si‐Dram1 cells, with retained autophagic dots and dispersed green fluorescence post‐rapamycin treatment, compared with effective clearance in si‐NC control cells. (D) ARS staining revealed significantly reduced osteogenic capacity in the si‐Dram1 group. Con, control; Dex, dexamethasone; Baf‐A1, bafilomycin A1; Rap, rapamycin; Rap/Rec, 8 h after rapamycin removal.

Our experiments demonstrate that long‐term, high‐dose dexamethasone significantly inhibits autophagic flux in MC3T3‐E1 cells. TEM analysis showed a marked reduction in the number of autophagosomes in dexamethasone‐treated cells compared with controls. Addition of Bafilomycin A1 (Baf‐A1) increased autophagosome numbers in both groups, but the increase was more pronounced in control cells, confirming dexamethasone's inhibitory effect on autophagic flux (Figure 5A).

Autophagosome clearance efficiency is another key indicator of autophagic flux. Immunofluorescence analysis showed that DRAM1 is predominantly localized to lysosomes (LAMP2) (Figure S5A).^23^ In control cells (si‐NC), rapamycin‐induced autophagy significantly increased LC3‐II levels, which nearly returned to baseline 8 h after rapamycin removal. However, in cells with Dram1 knockdown (si‐Dram1), LC3‐II levels remained elevated, suggesting impaired autophagic flux (Figure 5B). To further investigate the role of DRAM1 in autophagic flux under dexamethasone stimulation, cells were infected with a GFP‐TurboRFP‐LC3 dual fluorescence marker lentivirus. After rapamycin treatment, control cells (si‐NC) exhibited a gradual quenching of green fluorescence and an increase in autophagic dots, with the merged fluorescence appearing ‘warm’. In contrast, si‐Dram1 cells showed dispersed green fluorescence and fewer autophagic dots, appearing ‘cold’. Eight hours after rapamycin removal, control cells (si‐NC) displayed a significant decrease in autophagic dots, whereas si‐Dram1 cells showed a less pronounced decrease and retained a higher number of dots, indicating that Dram1 knockdown impairs autophagosome clearance in MC3T3‐E1 cells (Figure 5C). Relative quantification of ARS staining intensity demonstrated a significant reduction in mineralization capacity in the si‐Dram1 group, which may be attributed to the substantial inhibition of autophagic flux regulated by DRAM1 (Figure 5D).

### 3.6 Regulation of osteogenic differentiation by DRAM1 through the Wnt/β‐catenin signalling pathway

**FIGURE 6:**
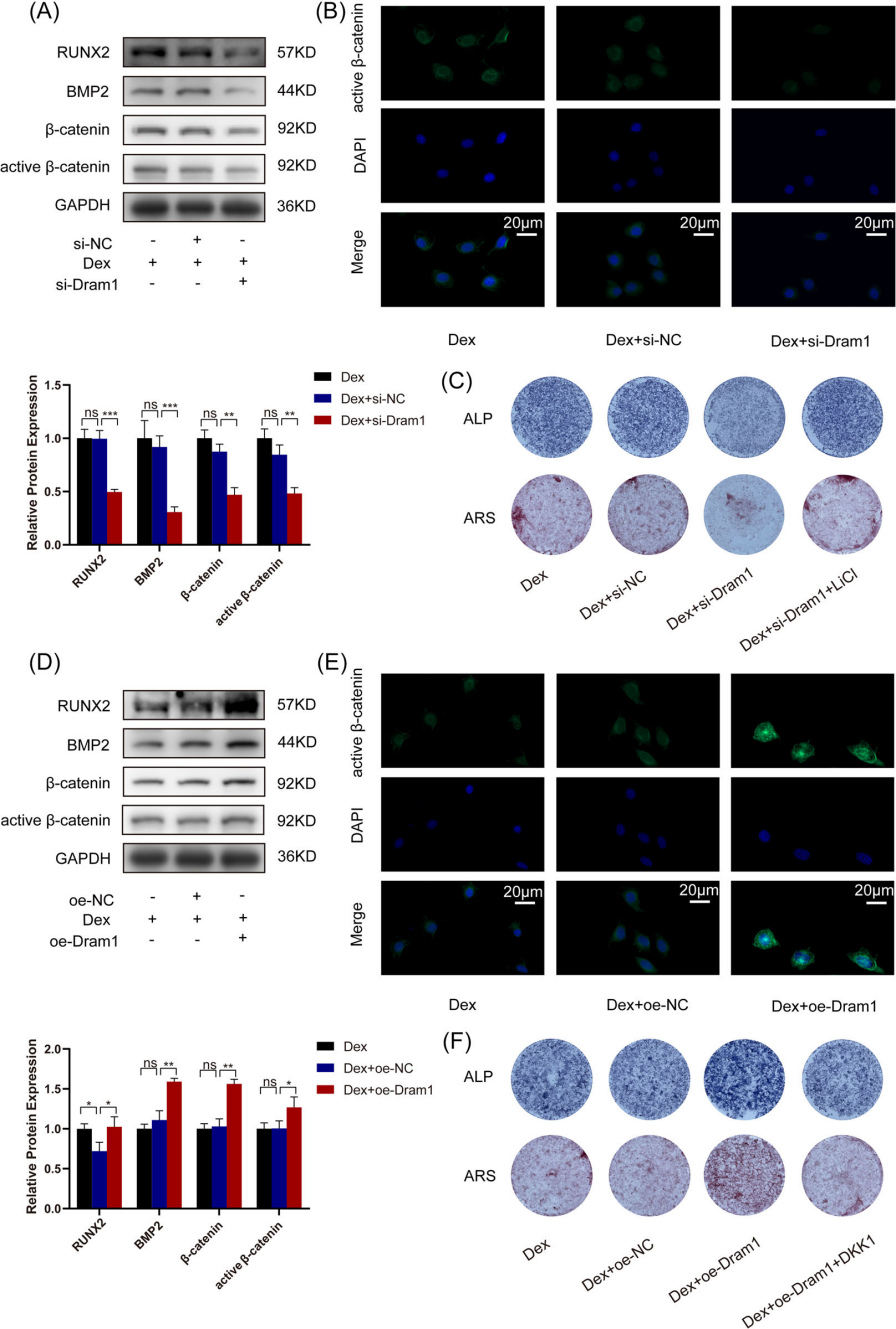
DRAM1 regulates osteogenic differentiation via the Wnt/β‐catenin signalling pathway in MC3T3‐E1 cells under high‐dose dexamethasone treatment. (A) Western blot showed reduced RUNX2, BMP2, β‐catenin and active β‐catenin levels in si‐Dram1‐transfected cells. (B) Immunofluorescence indicated decreased nuclear active β‐catenin in Dram1‐knockdown cells. (C) ARS and ALP staining showed reduced mineralization in Dram1‐knockdown cells, with partial recovery by LiCl. (D) Western blot revealed increased RUNX2, BMP2, β‐catenin and active β‐catenin levels in Dram1‐overexpressing cells. (E) Immunofluorescence showed elevated nuclear active β‐catenin in Dram1‐overexpressing cells. (F) ARS and ALP staining demonstrated enhanced mineralization in Dram1‐overexpressing cells, inhibited by DKK1. Dex, dexamethasone; DKK1, Dickkopf‐1.

Our experimental results indicate that, under prolonged high‐dose dexamethasone treatment, modulation of Dram1 expression in MC3T3‐E1 cells leads to significant changes in active β‐catenin expression. Based on these findings, we hypothesized that DRAM1 may regulate osteogenic differentiation through the Wnt/β‐catenin signalling pathway. In dexamethasone‐treated MC3T3‐E1 cells, transfection with si‐Dram1 significantly reduced the protein levels of RUNX2, BMP2, β‐catenin and active β‐catenin, as confirmed by western blot analysis (Figure 6A). Immunofluorescence analysis further showed a marked decrease in nuclear active β‐catenin in Dram1‐knockdown cells (Figure 6B). Furthermore, ARS and ALP staining indicated a significant reduction in mineralization capacity in Dram1‐knockdown cells, which was partially restored by LiCl, a Wnt/β‐catenin pathway activator (Figure 6C).^40^

Conversely, in Dram1‐overexpressing cells, western blot analysis showed a significant increase in RUNX2, BMP2, β‐catenin and active β‐catenin levels (Figure 6D). Immunofluorescence analysis also revealed elevated nuclear active β‐catenin (Figure 6E). ARS and ALP staining demonstrated enhanced mineralization capacity in Dram1‐overexpressing cells, which was significantly inhibited by Dickkopf‐1 (DKK1), a potent Wnt antagonist (Figure 6F).^40^ These findings suggest that DRAM1 regulates osteogenic differentiation through the Wnt/β‐catenin signalling pathway under prolonged high‐dose dexamethasone stimulation.

### 3.7 Animal experiments based on dexamethasone treatment

To confirm the mechanisms by which dexamethasone induces bone damage in vivo, we utilized a dexamethasone‐induced osteoporosis mouse model and evaluated the effects of resveratrol co‐treatment.^41^ Previous studies have shown that resveratrol can mitigate glucocorticoid‐induced bone damage in zebrafish.^42^ Likewise, other studies have demonstrated that resveratrol promotes osteogenic differentiation and protects murine induced pluripotent stem cells (iPSCs) from dexamethasone‐induced damage.^43^ The protective effects of resveratrol in the context of dexamethasone are closely associated with the activation of autophagy.^8^

**FIGURE 7:**
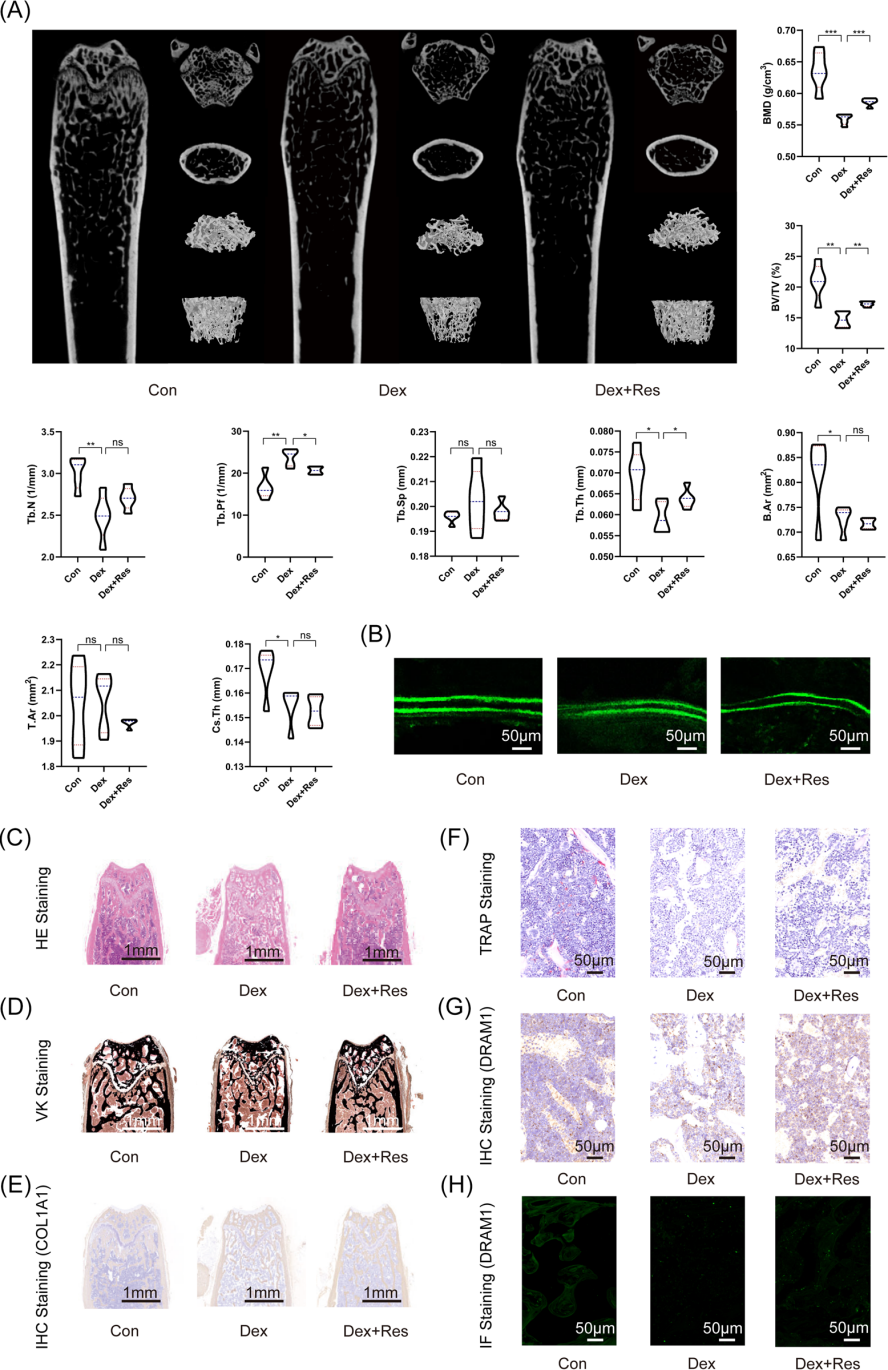
Resveratrol counteracts dexamethasone‐induced bone damage and enhances DRAM1 expression. (A) Micro‐CT analysis showed that dexamethasone decreased BMD, BV/TV, Tb.N, Tb.Th, B.Ar and Cs.Th, while increasing Tb.Pf. Resveratrol partially reversed bone damage. (B–E) Histological confirmation with calcein labelling, HE, VK staining and COL1A1 IHC supported micro‐CT findings. (F) TRAP staining revealed that dexamethasone inhibited osteoclast activity. (G, H) DRAM1 immunohistochemistry and immunofluorescence indicated that dexamethasone reduced DRAM1 expression, while resveratrol increased it. Con, control; Dex, dexamethasone; Res, resveratrol.

Micro‐CT analysis showed significant decreases in femoral BMD, BV/TV, Tb.N, Tb.Th, B.Ar and Cs.Th, along with an increase in Tb.Pf in dexamethasone‐treated mice, while no significant changes were observed in Tb.Sp or T.Ar. Resveratrol co‐treatment partially reversed the changes in BMD, BV/TV, Tb.Th and Tb.Pf (Figure 7A). These findings were supported by calcein double labelling, HE staining, VK staining, and COL1A1 immunohistochemistry (Figure 7B–E). TRAP staining indicated that prolonged high‐dose dexamethasone inhibited osteoclast activity, consistent with previous reports^44^ (Figure 7F). Immunohistochemistry and immunofluorescence analyses revealed reduced DRAM1 expression in the dexamethasone group, which was increased following resveratrol treatment (Figure 7G,H). This effect may be associated with resveratrol‐induced activation of autophagy.^45^ A similar effect of resveratrol on DRAM1 expression was observed in MC3T3‐E1 cells (Figure S6A). These results suggest that DRAM1 plays a crucial role in dexamethasone‐induced inhibition of osteogenesis.

## 4 DISCUSSION

Glucocorticoids, such as dexamethasone, are widely used in clinical practice but are known to induce osteoporosis by impairing osteogenic differentiation. This study demonstrates that dexamethasone affects osteogenic differentiation in a dose‐dependent manner. Low doses enhance osteogenic capacity in hBMSCs and MC3T3‐E1 cells, whereas prolonged high‐dose treatment inhibits differentiation and downregulates YTHDF1 and DRAM1 expression. These findings suggest that the dexamethasone‐induced inhibition of osteogenesis is closely linked to the regulation of YTHDF1 and DRAM1.

Further experiments showed that YTHDF1 controls the translation of DRAM1 mRNA via the m^6^A pathway. Dexamethasone disrupts this process by altering YTHDF1 expression and the methylation level of DRAM1 mRNA. MeRIP‐qPCR revealed a significant reduction in m^6^A modification of DRAM1 in hBMSCs under high‐dose dexamethasone treatment, while RIP‐seq analysis showed reduced enrichment of DRAM1 mRNA in sh‐YTHDF1 hBMSCs.^18^, ^46^ These results suggest that dexamethasone may regulate DRAM1 translation by affecting YTHDF1 expression and its recognition of DRAM1 m^6^A sites. Validation experiments in Ythdf1 KO and WT mice, together with in vitro studies examining YTHDF1 binding to intact or mutated m^6^A sites in Dram1, supported this hypothesis. In MC3T3‐E1 cells, overexpression of Ythdf1 increased DRAM1 protein levels and upregulated the osteogenic proteins RUNX2 and OCN, thereby enhancing osteogenic function, whereas Ythdf1 knockdown inhibited these effects.

Interestingly, although low‐dose dexamethasone treatment increases the protein expression of YTHDF1 and DRAM1, we speculate that this regulatory effect is not solely dependent on the canonical m^6^A‐mediated translational pathway. Our previous MeRIP‐seq data obtained from hBMSCs showed that the m^6^A methylation level of DRAM1 transcripts also decreased after treatment with 10^−9^ M dexamethasone for 3 days, consistent with the reduction observed under high‐dose, long‐term exposure.^18^ Therefore, it is possible that low‐dose dexamethasone promotes osteogenic differentiation through alternative mechanisms, such as enhanced transcription, involvement of other RNA‐binding proteins in translation, or increased post‐translational stability of DRAM1. These mechanisms warrant further investigation, as they may provide novel insights into the non‐canonical effects of glucocorticoids on osteogenesis.

In addition to m^6^A‐dependent regulation of DRAM1 by YTHDF1, our study further investigates the roles of autophagy and the Wnt/β‐catenin pathway, both mediated by DRAM1, in dexamethasone‐induced osteogenic inhibition. Autophagy and the Wnt/β‐catenin pathway are well recognized for their roles in osteogenic differentiation. Recent studies have highlighted the critical role of autophagy in GIOP. For example, Arbutin can alleviate glucocorticoid‐induced osteoporosis by activating autophagy in osteoblasts, thereby promoting osteoblast differentiation and mineralization while suppressing bone resorption.^47^, ^48^ Similarly, Galangin enhances osteogenic differentiation in GIOP models by activating autophagy through the PKA/CREB signalling axis, suggesting its potential as a therapeutic agent for GIOP.^49^ Additionally, Geniposide protects against dexamethasone‐induced osteoblast apoptosis by activating autophagy via the PI3K/AKT/mTOR pathway, further emphasizing the importance of autophagic regulation in bone homeostasis.^9^ Collectively, these studies indicate that activation of autophagy may counteract glucocorticoid‐induced bone loss, highlighting its therapeutic potential in GIOP. In parallel, the Wnt/β‐catenin pathway is vital for osteoblast activity and bone remodelling. Impairments in both autophagy and Wnt/β‐catenin signalling in GIOP models lead to reduced osteoblast differentiation and mineralization, thereby contributing to osteoporosis progression.

Our study systematically investigates the role of DRAM1 in regulating autophagy and the Wnt/β‐catenin signalling pathway under dexamethasone treatment, providing novel insights into the molecular mechanisms of glucocorticoid‐induced osteogenic inhibition. Under prolonged high‐dose dexamethasone exposure, DRAM1 expression significantly affected AKT/rpS6‐mediated autophagy and the level of active β‐catenin, thereby impairing osteogenic differentiation and mineralization. The AKT/rpS6 signalling pathway is an important regulator of autophagic activity. Our results suggest that DRAM1 may modulate autophagy, at least in part, via this pathway, as evidenced by decreased rpS6 phosphorylation and increased LC3‐II/I ratio upon Dram1 overexpression. Although the Wnt/β‐catenin pathway is primarily known for its pivotal role in osteoblast differentiation and bone remodelling, accumulating studies indicate its potential involvement in autophagy regulation.^50^ Particularly in the context of glucocorticoid‐induced bone loss, the interaction between Wnt/β‐catenin signalling and autophagy remains to be further investigated. In vivo experiments further suggested that DRAM1 may exert a protective role in dexamethasone‐induced osteoporosis, and demonstrated the potential therapeutic effect of resveratrol in this model.

Compared with previous studies, our research provides several key insights. First, we revealed a significant reduction in DRAM1 expression in the GIOP model, whereas prior studies reported increased DRAM expression in the OVX model.^24^ This differential expression likely reflects distinct mechanisms underlying bone loss in each model. OVX‐induced bone loss is primarily driven by estrogen deficiency, which may activate autophagy and upregulate DRAM1. In contrast, chronic glucocorticoid exposure in GIOP suppresses autophagy and impairs lysosomal function, potentially leading to reduced DRAM1 expression. In addition, species differences may contribute to this discrepancy, as previous OVX models were established in rats, whereas our GIOP model was established in mice, which could involve species‐specific regulatory responses. These factors may collectively account for the observed differences in DRAM1 expression between the two osteoporosis models. Second, we investigated the effects of glucocorticoids on the m^6^A modification of DRAM1 mRNA. Additionally, this study provides the first evidence that YTHDF1 specifically regulates DRAM1 expression via an m^6^A‐dependent mechanism. We also explored the impact of DRAM1‐mediated AKT/rpS6 signalling on autophagy, and examined the role of DRAM1‐regulated autophagic flux and the Wnt/β‐catenin pathway in osteogenesis. Finally, we demonstrated that the protective effect of resveratrol in osteoporosis may occur through DRAM1 activation. These findings provide new insights into the mechanisms of glucocorticoid‐induced osteoporosis and offer potential therapeutic strategies.

Despite these advances, our study has several limitations. First, the experiments were primarily conducted in mouse models and in vitro cell lines, which may not fully reflect the complexity of human bone physiology. Second, the relatively small sample size may limit the statistical power of our findings. Finally, while we focused on the roles of YTHDF1 and DRAM1 in m^6^A methylation, autophagy and the Wnt/β‐catenin pathway, other potential regulatory mechanisms remain to be explored.

Future research should expand the analysis of human pathological samples to validate the roles of YTHDF1 and DRAM1 in dexamethasone‐induced osteogenic inhibition and to assess their clinical relevance. Increasing the sample size would further improve the reliability and generalizability of the findings. Further studies should also investigate the mechanisms of YTHDF1 and DRAM1 across various animal models and cell lines, explore additional regulatory factors and signalling pathways, and develop novel therapeutic strategies targeting these molecules for glucocorticoid‐induced osteoporosis. Given the pivotal role of methylation in this process, methylation‐targeting therapies may represent a promising avenue for intervention.^51^, ^52^, ^53^ Collectively, these efforts will enhance our understanding of dexamethasone's impact on osteogenic differentiation and contribute to more effective prevention and treatment strategies.^54^ Moreover, with the growing integration of medicine, bioengineering, and materials science, future studies may benefit from incorporating advanced materials and bio‐devices into osteoporosis‐related research.^55^, ^56^, ^57^

## AUTHOR CONTRIBUTIONS

Ze‐Yu Lu, Peng‐Bo Chen and Xin‐Feng Zheng contributed to the conception and design of the study. Data preparation and collection were performed by Ze‐Yu Lu, Qing‐Yin Xu, Muradil Mardan, Yue‐Hua Yang and Tao Liu. Data analysis was conducted by Peng‐Bo Chen, Wen‐Ning Xu and Huo‐Liang Zheng. Hao Cai, Hui Deng, Xing‐Xu Huang and Bo Li were responsible for accessing and validating the underlying data. Reconciliation of the underlying data was performed by Sheng‐Dan Jiang and Lei‐Sheng Jiang. The draft of the manuscript was written by Ze‐Yu Lu and Peng‐Bo Chen. All authors reviewed and commented on previous versions of the manuscript. All authors read and approved the final manuscript.

## ETHICS STATEMENT

The experimental protocols involving human specimens and animals were approved by the Institutional Review Board (IRB) of Xinhua Hospital Affiliated to Shanghai Jiao Tong University School of Medicine (XHEC‐NSFC‐2019‐153).

## CONFLICT OF INTEREST STATEMENT

The authors declare no conflict of interest.

## Supporting information



Supporting Information

Supporting Information

## Data Availability

The data supporting the findings of this study are available from the corresponding author upon reasonable request.
