# Supplementary material for: m6A‐modified DRAM1 recognized by YTHDF1 regulates autophagy during dexamethasone‐induced osteogenic inhibition
Source: Clin Transl Med. 2026 Jul 22;16(7):e70655. doi: 10.1002/ctm2.70655 (PMC13392500; doi:10.1002/ctm2.70655)
Supplement: Supplementary file 1 — Supporting Information [file CTM2-16-e70655-s002.docx]

**Supplementary Figure 1**

**
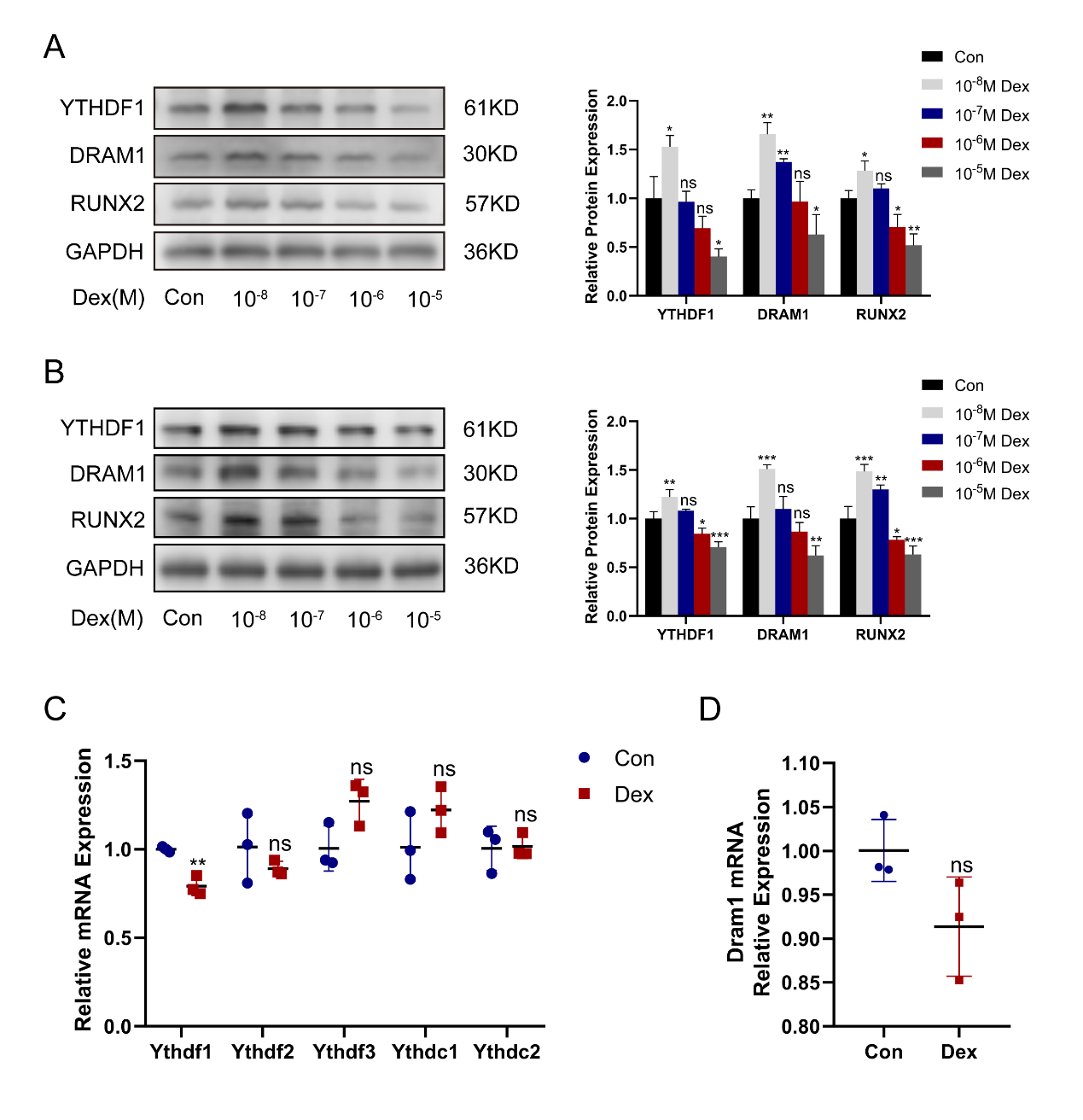
Supplementary Figure 1. Dose-dependent effects of dexamethasone on YTHDF1 and DRAM1 protein expression, and mRNA level changes after dexamethasone treatment.** **(A)** Western blot analysis of hBMSCs showed that dexamethasone at concentrations below 10^-7^ M increased the expression of YTHDF1, DRAM1, and RUNX2, whereas higher concentrations reduced their expression. **(B)** Western blot analysis of MC3T3-E1 cells showed a similar dose-dependent effect. **(C)** RT-qPCR analysis in MC3T3-E1 cells showed that 10^-5^M dexamethasone significantly reduced Ythdf1 mRNA expression, with no marked changes in Ythdf2, Ythdf3, Ythdc1, or Ythdc2. **(D)** RT-qPCR analysis in MC3T3-E1 cells showed no significant change in Dram1 mRNA levels after treatment with 10^-5^ M dexamethasone. (Abbreviations: Con, control; Dex, dexamethasone.)

**Supplementary Figure 2**

**
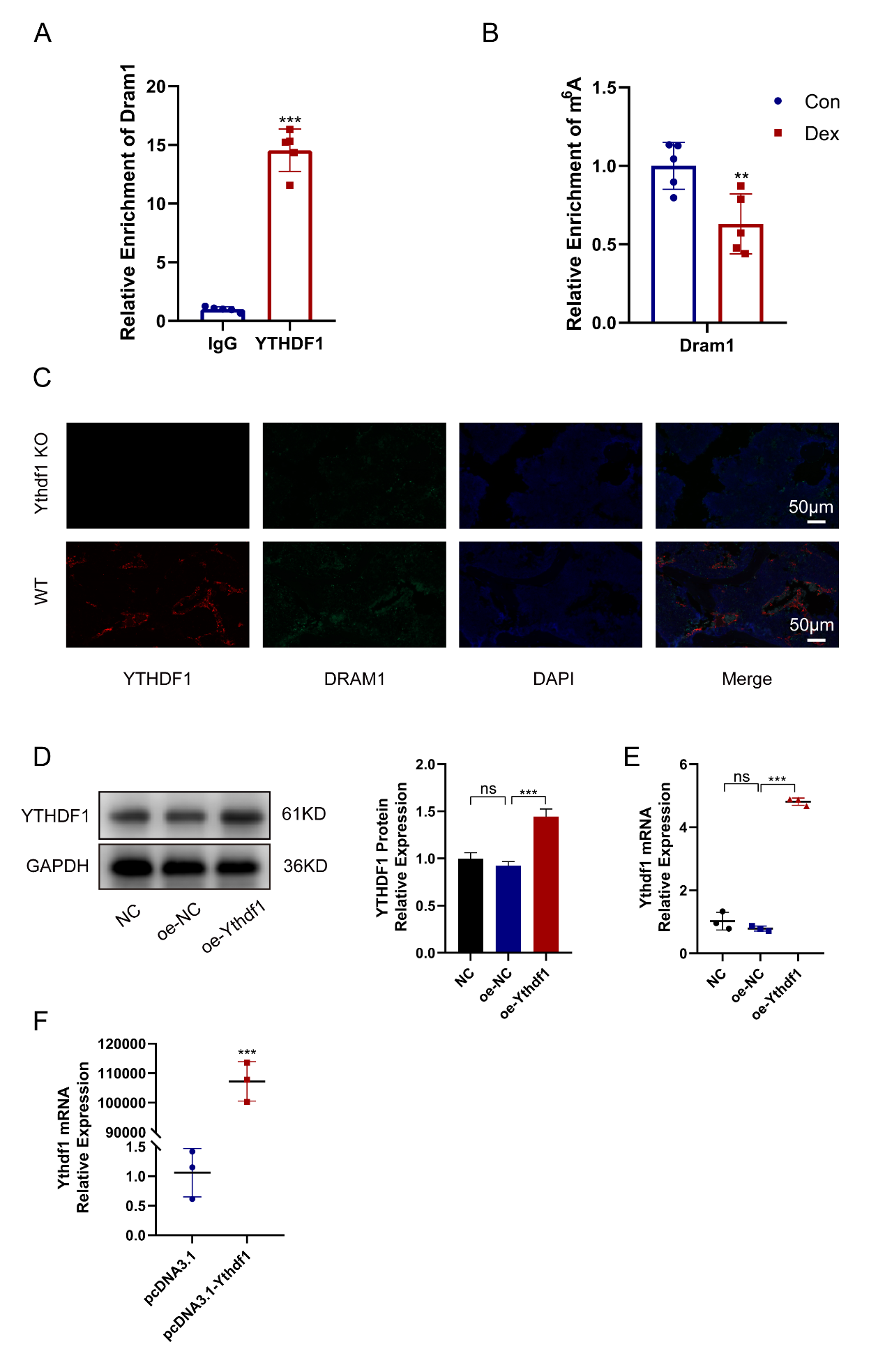
Supplementary Figure 2. Validation of the YTHDF1–Dram1 interaction in vivo and Ythdf1 overexpression efficiency.** **(A)** RIP-qPCR showed significant enrichment of Dram1 mRNA by anti-YTHDF1 antibody in WT mouse femurs compared with the IgG control. **(B)** MeRIP-qPCR revealed reduced m^6^A enrichment on Dram1 mRNA in femurs from mice treated with long-term intraperitoneal dexamethasone. **(C)** Immunofluorescence confirmed reduced DRAM1 expression in femoral tissue of Ythdf1 KO mice compared with WT mice. **(D,E)** Western blot and RT-qPCR analyses validated the effectiveness of Ythdf1 overexpression lentivirus in MC3T3-E1 cells. **(F)** RT-qPCR analysis confirmed successful overexpression of Ythdf1 in HEK-293T cells. (Abbreviations: Con, control; Dex, dexamethasone; m^6^A, N^6^-methyladenosine; WT, wild type; KO, knockout.)

**Supplementary Figure 3**

**
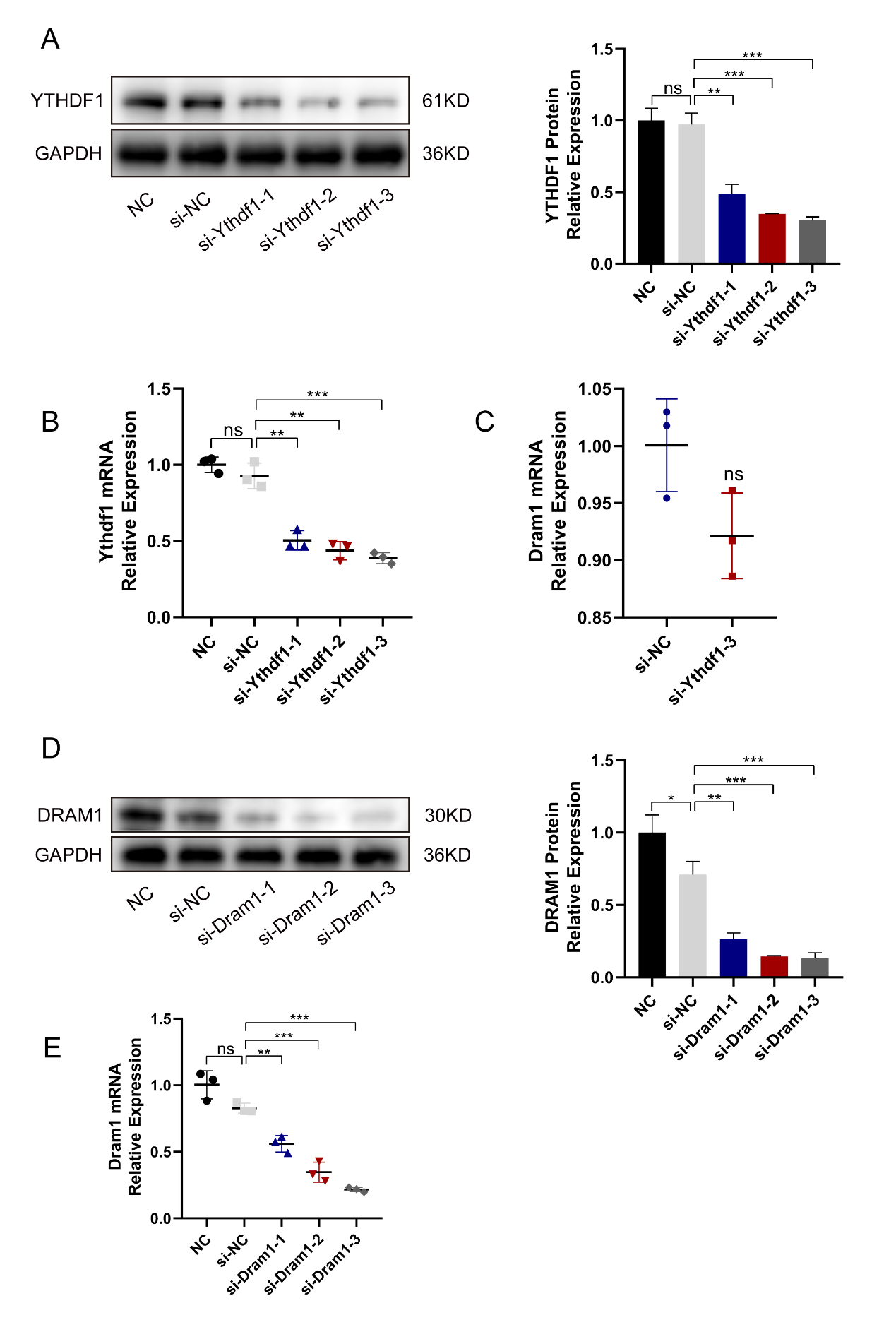
Supplementary Figure 3. Validation of siRNA efficiency for Ythdf1 and Dram1 in MC3T3-E1 cells.** **(A,B)** Efficiency of Ythdf1 siRNAs assessed by Western blot and RT-qPCR, with si-Ythdf1-3 showing the most effective knockdown. **(C)** RT-qPCR showed no significant change in Dram1 mRNA levels between si-Ythdf1-3 and si-NC groups. **(D,E)** Efficiency of Dram1 siRNAs evaluated by Western blot and RT-qPCR, with si-Dram1-3 demonstrating the most effective knockdown.

**Supplementary Figure 4**

**
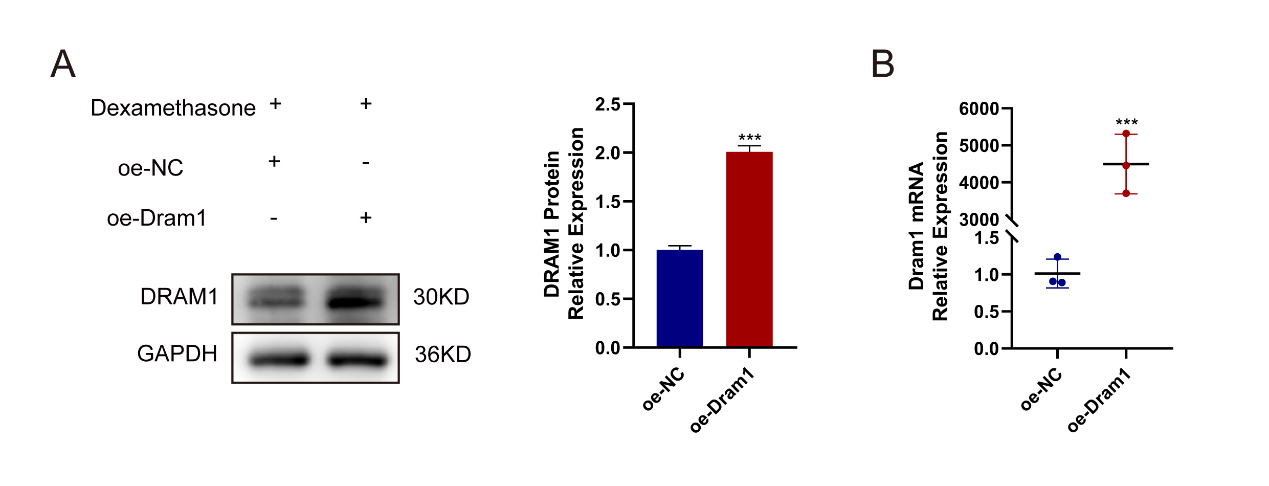
Supplementary Figure 4. Validation of DRAM1 overexpression effects.** **(A,B)** Western blot and RT-qPCR analyses confirmed increased DRAM1 protein expression and Dram1 mRNA levels in MC3T3-E1 cells transfected with a Dram1 overexpression plasmid.

**Supplementary Figure 5**

**
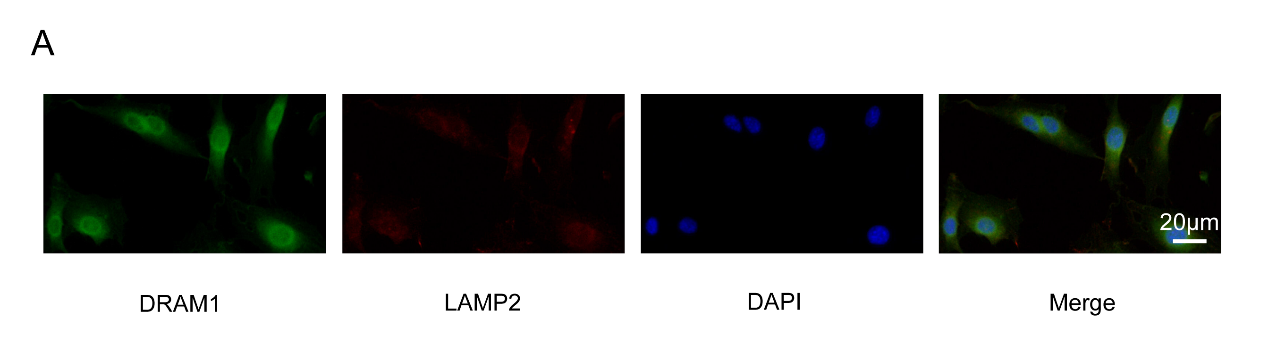
Supplementary Figure 5. Immunofluorescence analysis of DRAM1 localization in MC3T3-E1 cells. (A)** Cells were stained with antibodies against LAMP2 (red) to label lysosomes, and DRAM1 (green). The merged images showed colocalization of DRAM1 with lysosomes, indicating predominant localization of DRAM1 in lysosomal compartments. Nuclei were counterstained with DAPI.

**Supplementary Figure 6**

**
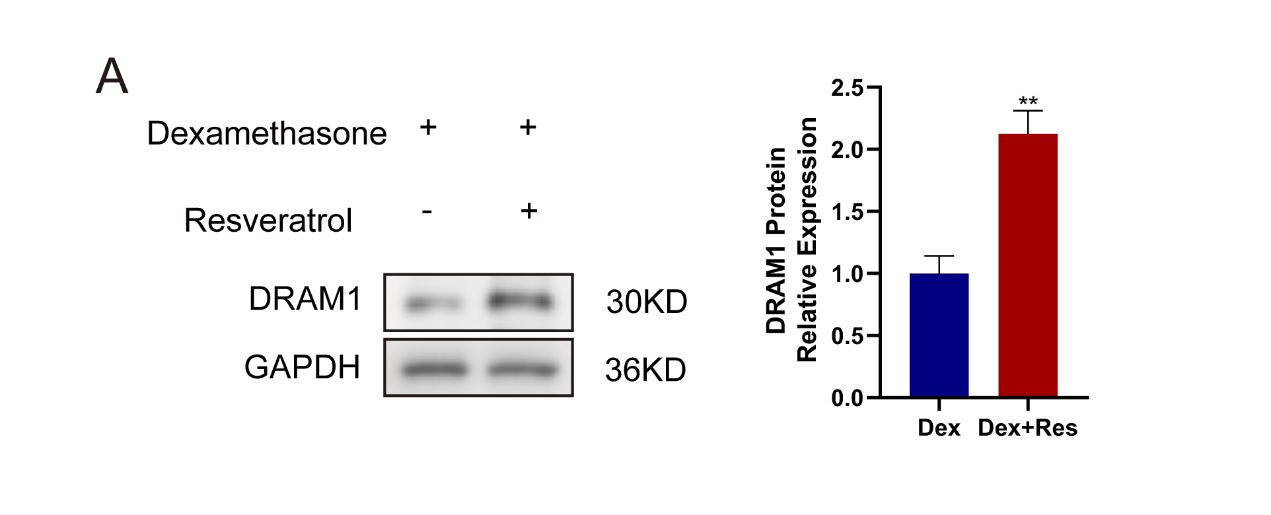
****Supplementary Figure 6. Analysis of resveratrol-mediated regulation of DRAM1 expression in MC3T3-E1 cells. (A)** Western blot analysis demonstrated that co-treatment with dexamethasone and resveratrol significantly upregulated DRAM1 protein levels compared with dexamethasone treatment alone. (Abbreviations: Dex, dexamethasone; Res, resveratrol.)
