# Supplementary material for: m6A‐modified DRAM1 recognized by YTHDF1 regulates autophagy during dexamethasone‐induced osteogenic inhibition
Source: Clin Transl Med. 2026 Jul 22;16(7):e70655. doi: 10.1002/ctm2.70655 (PMC13392500; doi:10.1002/ctm2.70655)
Supplement: Supplementary file 2 — Supporting Information [file CTM2-16-e70655-s001.docx]

**Supplementary Table 1.** Primer sequences for RT-qPCR.

| **Gene** | **Primer Sequences** |
| --- | --- |
| Human GAPDH | F: 5’-AGGTCGGTGTGAACGGATTTG-3’  R: 5’-GGGGTCGTTGATGGCAACA-3’ |
| Human DRAM1 | F: 5’-AGCCGCCTTCATTATCTCCT-3’  R: 5’-CACCAAGAAATGCAGAGAAGTT-3’ |
| Mouse Gapdh | F: 5’-AGGTCGGTGTGAACGGATTTG-3’  R: 5’-TGTAGACCATGTAGTTGAGGTCA-3’ |
| Mouse Ythdf1 | F: 5’-ACAGTTACCCCTCGATGAGTG-3’  R: 5’-GGTAGTGAGATACGGGATGGGA-3’ |
| Mouse Ythdf2 | F: 5’-AGGCGGGTTCTGGATCTACT-3’  R: 5’-GATAGGCGGCATCCAGTCTC-3’ |
| Mouse Ythdf3 | F: 5’-CAGAGACCTAAAGGGCAAGGA-3’  R: 5’-CATGCTGCTTCCCCAAGAGA-3’ |
| Mouse Ythdc1 | F: 5’-CGTAGGAAGCTGAGTGGAGC-3’  R: 5’-TCCCCATCTTTCTCCTCCCG-3’ |
| Mouse Ythdc2 | F: 5’-GGTCCGATCAATCATCTGT-3’  R: 5’-GAAGTAACGAATAGGCATGT-3’ |
| Mouse Dram1 | F: 5’-CCTGTCTCATTCAGGGAGTGT-3’  R: 5’-TGTCGTTGGTGCTATCCATCC-3’ |

**Supplementary Table 2.** Primer sequences for PCR.

| **Gene** | **Primer Sequences** |
| --- | --- |
| Mouse Ythdf1 | F: 5’-CACCTGAGTTCAGATCATTAC-3’  R: 5’-GCTCCAGACTGTTCATCC-3’ |
